# New Adrenaline Devices for Treating Anaphylaxis: Results of a Joint Survey From the European Anaphylaxis Registry and the Allergy‐Vigilance Network

**DOI:** 10.1002/clt2.70162

**Published:** 2026-03-07

**Authors:** Guillaume Pouessel, Sabine Dölle‐Bierke, Lea Faust, Dominique Sabouraud‐Leclerc, Yasemin Karaca‐Altintas, Margitta Worm

**Affiliations:** ^1^ Department of Pediatrics CH Roubaix Children's Hospital Roubaix France; ^2^ CHU Lille Pediatric Pulmonology and Allergy Department, Pôle Enfant Hôpital Jeanne de Flandre Lille France; ^3^ Univ Lille ULR 2694: METRICS Lille France; ^4^ Allergy‐Vigilance Network Vandoeuvre‐lès‐Nancy France; ^5^ Division of Allergy and Immunology Department of Dermatology, Venereology and Allergology Charité—Universitätsmedizin Berlin Corporate Member of Freie Universität Berlin Humboldt‐Universität zu Berlin Berlin Institute of Health Berlin Germany; ^6^ General and Specialized Pediatrics Department American Hospital Reims University Hospital Center Reims France; ^7^ University of Lille, CNRS, Inserm, CHU Lille Institut Pasteur de Lille U1019‐UMR 9017‐CIIL‐Center for Infection and Immunity of Lille Lille France

**Keywords:** adrenaline, allergy, anaphylaxis, auto‐injector, nasal

## Abstract

**Background:**

Adrenaline auto‐injectors (AAI) are underused to treat anaphylaxis. New adrenaline devices are currently under investigation or have been recently marketed. This survey aimed to assess the perspectives from allergy‐trained physicians regarding the AAI use and their expectations about new adrenaline devices.

**Methods:**

This electronic survey was created by the European Anaphylaxis Registry and Allergy‐Vigilance Network. It was proposed to their participants (March–April 2025) who were asked to rank their responses on a 11‐point Likert scale (0: ‘not important’ to 10: ‘very important’). Results are presented as median with interquartile range.

**Results:**

One hundred and seventy‐five physicians (allergists, 59.4%) participated in this survey. There were only few barriers to AAI prescriptions. Up to 65% of participants estimated the following features as very important for new adrenaline devices: prolonged shelf life (9 [7–10]), improved storage conditions (9 [5–9]), detailed pharmacokinetic‐pharmacodynamic data (8 [7–10]), optimised dose ranging (8 [7–10]), availability in public spaces (8 [7–10]), devices easy to carry (8 [7–9]), needle‐free device (8 [6–10]). A history of anaphylaxis treated with > 1 adrenaline injection (7 [4–9]) or admitted to intensive care unit (7 [3–8]) were reported as the most important barriers to use new adrenaline devices. 75% of participants felt that recommendations from allergy societies and more clinical data are important measures to reduce barriers to new adrenaline devices.

**Conclusions:**

Our data provide insights from allergy‐trained physicians into AAI limitations and expectations on new adrenaline devices. To advance them, input from allergy societies and more clinical data from anaphylaxis patients are needed.

**Abbreviations**
AAI: Adrenaline auto‐injector
AVN: Allergy‐vigilance network
IM: Intramuscular
IQR: Interquartile range
NORA: Network for Online Registration of Anaphylaxis
PK‐PD: Pharmacokinetic‐pharmacodynamic

## 1 Introduction

Adrenaline (epinephrine) is the first‐line therapy for anaphylaxis in all the recommendations [1, 2, 3]. The intramuscular route (IM) is recommended for the initial management of anaphylaxis in common practice. Adrenaline auto‐injectors (AAIs) allow a safe, quite ergonomic IM injection of adrenaline, at a suitable dosage, for everyone, including non‐professionals, in case of anaphylaxis. The international recommendations favour the use of these AAIs for the treatment of anaphylaxis in the community [1, 2, 3]. AAIs are widely present in the community despite ongoing disparities in global AAI availability among countries [4]. In England, prescriptions dispensed increased at an annual growth rate of around 9% over the past 20 years [5]. In France, approximately 1 million of AAIs were sold in 2022 [6].

Despite these recommendations, adrenaline and AAIs remain greatly underused for treating anaphylaxis in the community [7]. A delay in the injection of adrenaline is yet associated with a risk of worsening, refractory anaphylaxis as well as a risk for biphasic anaphylaxis [8, 9]. However, available data suggest that current AAIs may have only a little impact on preventing fatal anaphylaxis [5].

Certain issues and barriers have been raised with respect to the use of AAIs [10]. Thus, innovations enabling the use of alternative forms of adrenaline (through novel routes) are desirable and new forms of adrenaline administration, in particular by intranasal or sublingual route, are currently being studied or have been recently marketed [11]. In the United States, on March 2025, the Food and Drug Administration (FDA) approved Neffy ‘for the emergency treatment of allergic immediate reactions (Type I), including those that are life‐threatening (anaphylaxis), in adult and paediatric patients aged 4 years and older who weigh 33 lbs (15 kg) or greater.’ In Europe, on August 2024, the European Commission has also approved EURneffy for the emergency treatment of allergic reactions as a useful alternative to injectable forms of adrenaline in adults and children weighing 30 kg or more.

The aim of this survey was to assess the perspectives from allergists regarding the prescription and use of AAIs and expectations about new routes of adrenaline administration.

## 2 Methods

### 2.1 Online Survey

This survey has been created and validated by the joint scientific committees of the European Anaphylaxis Registry (Network for Online Registration of Anaphylaxis [NORA])) and the Allergy‐Vigilance Network (AVN). The NORA and AVN collect data on real‐life anaphylaxis from moderate to severe anaphylactic reactions with the collaboration of allergy‐trained physicians. The NORA was launched in 2007 in German‐speaking countries (Germany, Switzerland, Austria) and expanded to other countries (France, Italy, Poland, Spain, Ireland, Greece, Romania, Bulgaria and Brazil) from 2011. The AVN is a French‐speaking registry comprising approximately 300 allergists (adults and/or paediatric allergists), collecting data since 2002 and collaborating with the NORA.

The online version of the questionnaire has been made available through the REDCap (Research Electronic Data Capture) database system, beta‐tested and launched during the 8th International conference of the NORA (March 28th–29th, 2025 Berlin, 43 participants). The 5‐min survey has been proposed to additional allergy‐trained participants, collaborating with both networks (AVN, *n* = 184; NORA, *n* = 50), during 1 month with three electronic reminders. The survey was originally written in English and translated into French for French‐speaking participants.

The survey consisted of questions in 4 main sections using 12 questions (See Supplementary information): Part 1. Demographic data of the participants (Questions 1–5); Part 2. Experience about AAI use and barriers to AAI prescription (Questions 6–7); Part 3. Expectations and potential barriers to prescribe and use new adrenaline devices (Questions 8–11); Part 4. Potential impact of new adrenaline devices at the community level (Question 12). Participants were asked to rate their response on a 11‐point Likert scale, from 0 (‘very difficult to use’ or ‘not all important,’ or ‘strongly disagree’ or ‘no impact’) to 10 (‘very easy to use’ or ‘very important,’ ‘strongly agree’ or ‘high impact’) depending on the type of questions (sections 2, 3, 4). The 11‐point Likert scale survey gives a much broader spread of the options to the participants compared to other Likert scales and yields clear indicative results. As for interpretation, we ranked the responses as ‘very important’ or ‘high impact’ (8–10), as ‘not important’ or ‘no impact’ (0–3) and as ‘neutral’ (4–7).

All responses were anonymous and volunteer. Only one response per participant was permitted.

### 2.2 Statistical Analysis

Statistical analyses were performed with SPSS Software (IBM SPSS Statistics, Version 25). The normality of distribution was verified for all items of the questionnaire with Kolmogorov‐Smirnov tests. This was an exploratory analysis. The survey has been submitted to allergy‐trained participants collaborating with NORA and AVN to a total of 277 physicians (sample size) The margin of error accepted was 5%, the confidence level 95% and the response distribution rate 50%. With these assumptions, the minimum number of respondents needed was 162 respondents. We received 175 responses.

As the responses to items do not follow a normal distribution, these variables were expressed as median and interquartile range (IQR). Figures represent violin plots of the distributions of responses for each item which were realised with GraphPad Prism software (version 8.0.0, San Diego, California USA).

To compare responses according to countries or medical specialities, distributions of responses for each item were compared using Kruskal‐Wallis tests. Because of the multiplicity of tests, the significance for each item was adjusted using Bonferroni correction. Since 41 tests were simultaneously performed to compare responses to items by speciality or country, each test was considered significant if *p*‐value was below 0.002 after rounding. For the others tests not requiring adjustment, *p*‐value below 0.05 was considered as significant.

## 3 Results

### 3.1 Demographic Data of the Participants

A total of 175 responses (response rate: 63%), mostly from Europe (France, *n* = 87 [50%]; Austria, *n* = 39 [22%]; Germany, *n* = 20 [11%]), were analysed (Table 1). Participants were mainly allergists (including paediatric allergists) (*n* = 104, 59.4%) and paediatricians (*n* = 64, 36.6%) with a median time of practice of 12 years (IQR: 4–27) and 70% were active members of the NORA and/or the AVN.

**TABLE 1:** Baseline characteristics.

Characteristic	*N* = 175^a^
Country	
Austria	39 (22%)
Belgium	5 (2.9%)
France	87 (50%)
Germany	20 (11%)
Italy	4 (2.3%)
Poland	5 (2.9%)
Spain	4 (2.3%)
Switzerland	2 (1.1%)
Other: Algeria, Bulgaria, Croatia, Ireland, Luxembourg, Macedonia, Morocco, Netherlands, Romania	*N* = 1, each 9 (4.5%)
Medical speciality	
Allergology (including pediatric allergology)	104 (59.4%)
Dermatology	18 (10.6%)
Internal medicine	5 (2.9%)
Otorhinolaryngology	1 (0.6%)
Paediatrics	64 (36.6%)
Pneumology	13 (7.6%)
Years of practice^b^	12 (4.27)
Sex, female	126 (72%)
NORA/AVN members	123 (70%)

Abbreviations: AAI, adrenaline auto‐injector; AVN, allergy‐vigilance network; NORA, network for online‐registration of anaphylaxis.

^a^ *n* (%).

^b^ Median (IQR).

### 3.2 Experience About AAI Use and Barriers to AAI Prescription

Of the 175 responders, 128 (73%) had ever used AAI, 71 (55%) less than 10 times and 38 (30%) more than 100 times.

The user‐friendliness of AAI was rated as quite easy to use, with a median value of 8 (IQR: 7–9) from the participant's experience and as moderately easy to use from the patient's perspective, with a median value of 6 (IQR: 5–7).

**FIGURE 1:**
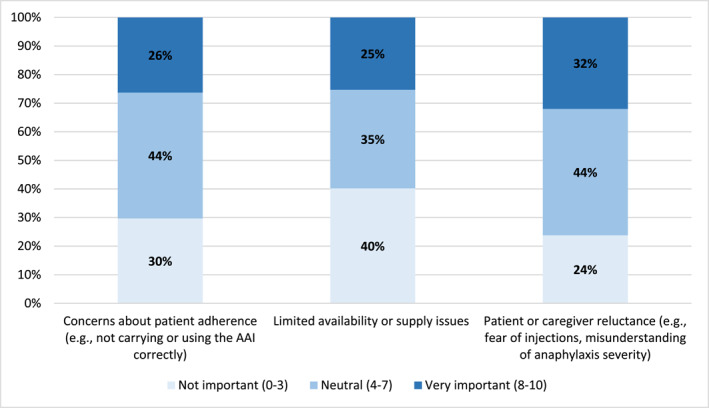
Adrenaline auto‐injector's satisfaction with current situations. The results are presented in bars for each item with the distribution according to the percentage of responses ranked from ‘not important’ (0–3), ‘neutral’ (4–7), ‘very important’ (8–10).

The main barriers to prescribe an AAI were concerns about patient adherence (median: 6 [IQR: 3–8]) and patient‐caregiver reluctance (e.g., fear of injections, misunderstanding of anaphylaxis severity) (median: 6 [IQR: 2–8]), indicating neutral to moderately important barriers (Figure 1, Supporting Information S1: Table S1). On the opposite, high cost/lack of insurance coverage (median: 1 [IQR: 0–5]) and uncertainty about the indication of AAI (median: 3 [1, 2, 3, 4, 5, 6, 7]) were not of important barriers to AAI prescription, respectively (Table S1).

### 3.3 Expectations and Potential Barriers to Prescribe and Use New Adrenaline Devices

**FIGURE 2:**
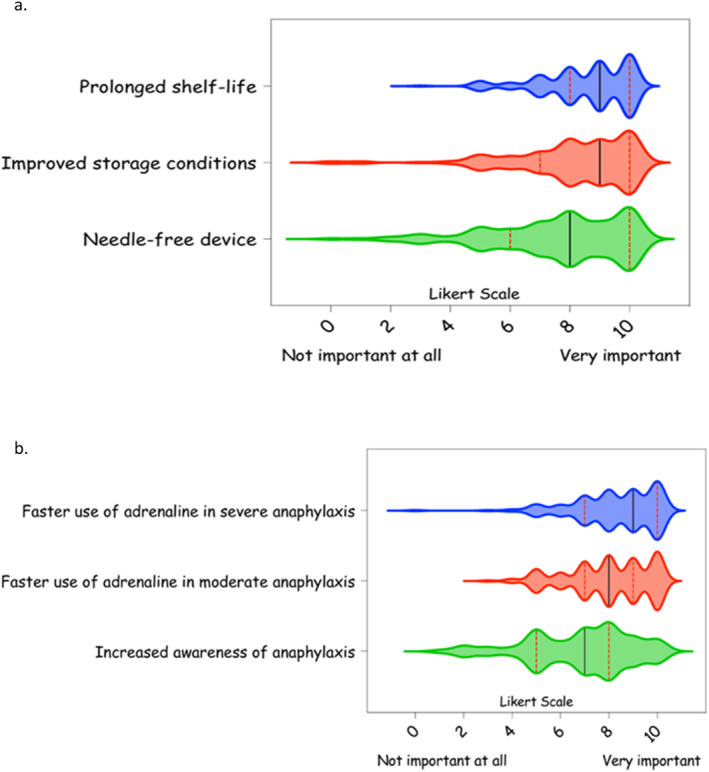
New adrenaline devices: a. Main expectations; b. Potential impact at the community level. The responses are presented with violin plots (indicating median value and interquartile range) on a Likert scale from 0 (‘not important at all’) to 10 (‘very important’).

The following features were regarded to be very important for new adrenaline devices compared to AAI: prolonged shelf life (138 participants [79%], median: 9 [IQR: 7–10]), improved storage conditions (*n* = 128 [73%], median: 9 [IQR: 5–9]), detailed pharmacokinetic‐pharmacodynamic (PK‐PD) data (*n* = 114 [65%], median: 8 [IQR: 7–10), optimised dose ranging (*n* = 121 [69%], median: 8 [IQR: 7–10]), availability in public spaces (*n* = 117 [67%], median: 8 [IQR: 7–10]), devices easy to carry (*n* = 117 [67%], median: 8 [IQR: 7–9]), needle‐free device (*n* = 107 [61%], median: 8 [IQR: 6–10]) (Figure 2a, Table S1, Figure S1). On the opposite, 89 (51%) and 84 (48%) participants ranked as ‘neutral’ their expectation on the need to reduce cost (median: 7 [IQR: 5–9]) and size of these new adrenaline devices (median: 7 [IQR: 5–9]) (Table S1, Figure S1).

**FIGURE 3:**
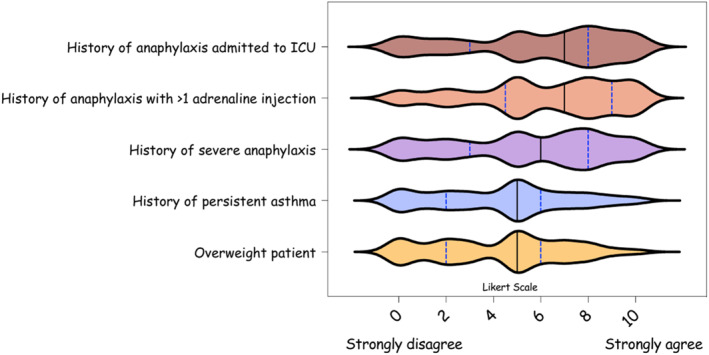
Main barriers to use any new nasal adrenaline device. The responses are presented with violin plots (indicating median value and interquartile range) on a Likert scale from 0 (“strongly disagree”) to 10 (“strongly agree”).

Main potential barriers to use any new intranasal adrenaline device were a history of anaphylaxis treated with more than one adrenaline injection (median: 7 [IQR: 4–9]) or intensive care unit admission (median: 7 [IQR: 3–8]) (Figure 3, Table S1, Figure S2). Reasons not to use new adrenaline devices of a less importance included: patient with obesity (*n* = 74 [42%], median: 5 [2, 3, 4, 5, 6]), history of a persistent asthma (*n* = 67 [38%], median: 5 [2, 3, 4, 5, 6]) and mastocytosis (*n* = 51 [29%], median: 5 [3, 4, 5, 6, 7, 8]) (Table S1, Figure S2).

One hundred and twelve out of 175 (64%) responders strongly agreed that the lack of clinical data about the new adrenaline device was a very important prescription barrier (median: 8 [IQR: 7–10]). 131 (75%) participants estimated that recommendations from allergy society (median: 9 [7.75–10]) and more clinical data (median: 9 [IQR: 7–10]) would be very important measures to help reducing barriers to a new adrenaline device (Table S1, Figure S3).

### 3.4 Potential Impact of New Adrenaline Devices at the Community Level

One hundred and twenty‐one out of 175 (69%) responders felt that new adrenaline devices would have a very important impact to increase the use of adrenaline to treat anaphylaxis in the community (median: 8 [IQR: 7–10]), a more prompt use of adrenaline in patients with severe and mild/moderate anaphylaxis (*n* = 124 [71%] and *n* = 114 [65%], median: 9 [IQR: 7–10] and 8 [7, 8, 9]), a wider access to adrenaline in public spaces (*n* = 100 [57%], median: 8 [IQR: 6–9]), a wider access to adrenaline in schools (*n* = 105 [60%], median: 8 [IQR: 7–10]) and a greater global availability of adrenaline (*n* = 109 [62%], median: 8 [IQR: 6.75–10]) (Figure 2b, Table S1, Figure S4).

### 3.5 Country Specific Considerations

We observed significant differences among countries for various items (Table S2). The rate of AAI user‐friendliness from participant's perspective was higher in France and Germany compared to Austria and other countries (*p* = 0.002). The high cost/lack of insurance coverage had less impact on AAI prescription in France, Germany and Austria compared to other countries (*p* = 0.001). Limited availability/supply issues was an important barrier to AAI prescription mainly in France compared to other countries (*p* < 0.001). Reduced size of the new adrenaline device was of a greater importance in Austria and Germany compared to France and other countries (*p* = 0.002).

The reasons not to use a new adrenaline device were ranked differently according to countries for the following items: overweight patient (*p* = 0.001), history of severe anaphylaxis (*p* = 0.001) or anaphylaxis admitted to intensive care unit (*p* = 0.001), history of persistent asthma (*p* < 0.001) or mastocytosis (*p* < 0.001).

### 3.6 Impact of Medical Specialities

The rate of the responses was significantly different according to medical specialities for only one item about limited availability or supply issues as a barrier to prescribe an AAI (Table S3). The median rate to this item was higher for allergists and physicians of other specialities compared to paediatricians and dermatologists (*p* = 0.002).

## 4 Discussion

This survey was designed to assess limitations and needs regarding AAI use as well as expectations about alternatives forms to injectable adrenaline for treating anaphylaxis from the perspectives of allergy‐trained physicians. In addition, this survey aimed to rank these features to highlight points of interest and unmet needs.

There were quite few barriers to AAI prescription from the physicians' perspective, mainly patient and caregiver reluctance to use the device (e.g., fear of injections or misunderstanding of anaphylaxis severity) and concerns about patient adherence (e.g., not carrying or using the AAI correctly), although the median ratings reached the neutral to moderate range of the scale. This observation is surprising considering that the medical prescription of an AAI should be independent from any presumed patient's adherence.

Despite few barriers to AAI prescription, physicians expressed great expectation on various aspects about new adrenaline devices: design and characteristics of devices (easy to carry, needle‐free prolonged shelf life, improved storage conditions, optimised dose ranging), and wider access in the public spaces. Yet, some of these features are perceived as potential limitations of AAI use from the physician's perspective but analysis of the literature does not support this perception. For example, there seems to be issues about adrenaline stability under extreme storage conditions and after freezing. However, in a systematic review of adrenaline degradation with exposure to extreme temperatures, studies that tested short exposure to extreme heat (< 24 h), or real‐world exposures, did not reveal any significant degradation [12]. In a survey conducted among 200 caregivers of paediatric patients with food allergies and designed to understand the factors associated with underuse of AAI, the reasons caregivers indicated for not administering the AAI were multiple but needle fear was reported by only 4% of the participants whereas issues about the lack of carriage of the device were not reported at all [13].

An easy to carry device is expected by participants from new adrenaline devices. Indeed, many studies reported that patients who have been prescribed AAIs after an anaphylactic event do not carry their devices with them [7]. For example, only 226/1538 (14.7%) self‐treated cases in the NORA received adrenaline via AAI [7]. In a further 22.4%, AAI was available but not used.

Effective prevention of severe anaphylaxis events requires a worldwide availability of AAIs. In this context, it should be remembered that AAIs are available in only certain countries in the world. In a study carried out by the World Allergy Organisation, only 60% of the 66 participating countries had access to AAIs [4]. Many countries in South America, Africa or certain regions in Asia have no access or depend on imports. In addition, repeated tensions in the supply of AAIs have also been reported over several years in the European market and lead to regular and random periods of breaks in pharmacy dispensing.

In our survey, physicians expressed the need for more detailed PK‐PD data to feel more confident before prescribing new adrenaline devices. In fact, despite number of studies performed in healthy adults and children designed to assess PK‐PD data on AAI and new adrenaline devices, there are currently insufficient PK‐PD data relative to the use of adrenaline during anaphylaxis [6, 14, 15, 16, 17, 18, 19]. Such limitations regarding PK‐PD data could be addressed through hospital challenges studies, in both paediatric and adult populations, but also using real‐world registry‐based outcome data, from emergency rooms and intensive care units. Up to now, the lack of PK‐PD data on AAI and new adrenaline devices in anaphylaxis patients may be a limitation to specific recommendation to the use of new adrenaline devices compared to AAI.

The reasons not to use a new adrenaline device included patients who experienced anaphylaxis requiring > 1 adrenaline injection or leading to admission to intensive care unit. These two conditions define severe anaphylaxis despite no clear clinical definition of this entity so far [20]. In 2019, the Anaphylaxis Committee of the World Allergy Organisation proposed that a ‘severe’ anaphylaxis is ‘characterized by potentially life‐threatening compromise in breathing and/or the circulation, which may occur without typical skin features or circulatory shock being present’ [21]. The responses of our survey suggest that intranasal adrenaline devices may be perceived by physicians as less effective or not enough proven in clinical practice compared to AAI in treating severe anaphylaxis. On the opposite, our data suggest that various medical conditions may not be barriers to use intranasal adrenaline devices, such as overweight patients, patients with a history of persistent asthma, mastocytosis or successfully treated with an AAI before. These findings reflect our inability to accurately predict the most at‐risk individuals of severe anaphylactic reactions, particularly those with food allergies [22]. Considering the impact of obesity, a review of PK‐PD data of 4 AAIs does not support that the body mass index is an important surrogate of an adequate adrenaline delivery [14]. Consistently, the majority of participants felt that recommendations from allergy societies and more clinical data would be valuable measures for reducing barriers to new adrenaline devices.

Our results highlight significant differences regarding issues about cost/lack of insurance coverage, limited availability/supply and reasons not to use new adrenaline devices among countries, but not among medical specialities. These differences of perception are easy to understand in mirror to disparities regarding AAI insurance coverage and cost among countries. For example, in 2024, a two‐AAI Epipen pack was commonly sold 600 USD in the United States, 85 USD in Germany, 76 USD in France 61 USD in the UK, and 30 USD in India [23]. The same disparities are reported in Europe for other brands. For example, in 2025, a 150 μg AAI Jext was commonly sold 29.14 Euros in Spain, 49.83 Euros in Switzerland, 44.47 Euros in Netherlands, 49.69 Euros in Italy, and 33.44 Euros in Belgium (manufacturer data).

In parallel to a great expectation on new adrenaline devices, most participants estimated that these devices could have a major impact on a faster use of adrenaline in patients with severe anaphylaxis, increased use in anaphylaxis in the community at all and a wider global access, including in schools. If an adrenaline device is carried all the time by the patient and used promptly at onset of first symptoms, needle‐free devices may be favoured by affected patients in particular in the community.

## 5 Limitations

We used a 11‐point Likert scale to better discriminate the responses. Such expanded range enables participants to communicate their perspectives with more prominent explicitness. A 11‐point scale considers a more nuanced comprehension of representative fulfilment, recognising minor varieties that may be missed with a shorter scale. However, to interpret the responses in another manner than with the median and IQR values, we decided to rank arbitrarily the responses as ‘very important’ or ‘high impact’ (8–10), as ‘not important’ or ‘no impact’ (0–3) and as ‘neutral’ (4–7). This classification is questionable, but we noticed that the distribution of the responses according to median and quartile values were in accordance with this classification. A limited number of physicians were included in the survey. Countries and subspecialities were not equally distributed.

Finally, the respondents may not be exactly representative to allergists in general or even non‐allergists (HCP) given their specialisation in the diagnosis and management of anaphylaxis. Moreover, the report of patient's perspectives is ideally derived from the patients themselves rather than their doctors as these might differ.

## 6 Conclusions

New forms of adrenaline administration, by intranasal, sublingual or transcutaneous route, are currently being studied and provide promising results to optimisecare. An intranasal adrenaline device has been marketed in the United States and recently in Germany. New adrenaline devices using alternative routes of administration can open the current arsenal of treatments available by addressing barriers and perceived limits to the current use of AAIs by physicians. Patient's perspectives are required regarding the use of AAI and their expectations about new routes of adrenaline administration. Considering the physicians expectations about the adrenaline use in anaphylaxis, we need to move forward to the following actionable steps: real‐world data on anaphylaxis and adrenaline treatment, promotion of stock adrenaline policies in public spaces and school settings, recommendations from international allergy societies to help guiding physicians on a stratified prescription approach.

## Author Contributions

**Guillaume Pouessel:** conceptualization, investigation, writing – original draft, methodology, validation, visualization, writing – review and editing, formal analysis, data curation, supervision, resources. **Sabine Dölle‐Bierke:** conceptualization, methodology, software, data curation, supervision, formal analysis, resources, validation, visualization, writing – review and editing, investigation. **Lea Faust:** conceptualization, methodology, software, data curation, formal analysis, writing – review and editing, validation, resources, visualization. **Dominique Sabouraud‐Leclerc:** investigation, validation, writing – review and editing, formal analysis, data curation, visualization. **Yasemin Karaca‐Altintas:** formal analysis, software, methodology, validation, visualization, writing – review and editing, writing – original draft, resources. **Margitta Worm:** conceptualization, investigation, writing – review and editing, visualization, validation, methodology, software, formal analysis, data curation, supervision, resources.

## Funding

This work was supported by NORA e.V.

## Conflicts of Interest

G.P. declares that he has received fees for scientific work or consulting requested by ALK, DVB Technologies, AI Therapeutics, Bioprojet, Theravia, Stallergenes, Viatris, Novartis. MW reports honoraria and/or consulting fees from AbbVie, Aimmune Therapeutics, ALK‐Abelló, Allergopharma, Almirall, Amgen, Biotest, Boehringer Ingelheim, DBV Technologies, Genzyme, Kymab, LEO Pharma, Eli Lilly, Mylan/Viatris, Novartis, Pfizer, Regeneron Pharmaceuticals Inc., Sanofi, Stallergenes Greer, Worg Pharmaceutics. Sabine Dölle‐Bierke, Dominique Sabouraud‐Leclerc, Yasemin Karaca‐Altintas, Lea Faust: The authors declare no conflicts of interest.

## Supporting information


Supporting Information S1

## Data Availability

The data that supports the findings of this study are available in the supplementary material of this article.
