# Supplementary material for: New Adrenaline Devices for Treating Anaphylaxis: Results of a Joint Survey From the European Anaphylaxis Registry and the Allergy‐Vigilance Network
Source: Clin Transl Allergy. 2026 Mar 7;16(3):e70162. doi: 10.1002/clt2.70162 (PMC12967222; doi:10.1002/clt2.70162)
Supplement: Supplementary file 1 — Supporting Information S1 [file CLT2-16-e70162-s001.docx]

**Supplementary information**

**New adrenaline devices for treating anaphylaxis: results of a joint survey from the European Anaphylaxis Registry and the Allergy-Vigilance Network.**

Table S1. Rank of responses (expressed as median, interquartile range and range) according to the 175 participants about limitations and needs regarding the use of adrenaline auto-injectors, and expectations about alternatives forms to injectable adrenaline for treating anaphylaxis.

|  | **Number**  **of responses** | **Median (IQR)** | **Range** |
| --- | --- | --- | --- |
| **Use of AAI** |  |  |  |
| How do you rate the user-friendliness of the AAI? | 128 | 8 (7-9) | 0-10 |
| How do you rate the user-friendliness of the AAI from the patient perspective? | 126 | 6 (5-7) | 0-10 |
| **What are the main barriers to prescribe an AAI in your practice?** |  |  |  |
| Uncertainty about the indication or need for an AAI | 174 | 3 (1-7) | 0-10 |
| High cost or lack of insurance coverage | 174 | 1 (0-5) | 0-10 |
| Concerns about patient adherence (e.g., not carrying or using the AAI correctly) | 175 | 6 (3-8) | 0-10 |
| Limited availability or supply issues | 174 | 5 (2-8) | 0-10 |
| Patient or caregiver reluctance (e.g., fear of injections, misunderstanding of anaphylaxis severity) | 172 | 6 (2-8) | 0-10 |
| **What are the most important features you expect from new adrenaline devices to treat anaphylaxis as compared to AAI?** |  |  |  |
| Detailed pharmacokinetic-pharmacodynamic data | 173 | 8 (7-10) | 1-10 |
| Optimised dosing with wide dose range (from infancy to elderly, overweight-obese individuals) | 175 | 8 (7-10) | 2-10 |
| Needle-free device | 175 | 8 (6-10) | 0-10 |
| Availability in public spaces (schools, restaurants, shopping precincts, airports and airplanes, sport stadia…) | 172 | 8 (7-10) | 0-10 |
| Reduced cost | 174 | 7 (5-9) | 0-10 |
| Improved storage conditions adapted to real life conditions | 173 | 9 (5-9) | 0-10 |
| Prolonged shelf-life | 172 | 9 (7-10) | 3-10 |
| Reduced size of the device | 174 | 7 (5-9) | 0-10 |
| Easy to carry | 175 | 8 (7-9) | 1-10 |
| **A new adrenaline device (by nasal) is available with comparable pharmacokinetic-pharmacodynamic data to AAI and a marketing authorization**  **What would be reasons not to use the new device?** |  |  |  |
| Overweight patient | 171 | 5 (2-6) | 0-10 |
| History of severe anaphylaxis | 174 | 6 (3-8) | 0-10 |
| History of anaphylaxis admitted to intensive care unit | 174 | 7 (3-8) | 0-10 |
| History of anaphylaxis treated with more than one adrenaline injection | 173 | 7 (4-9) | 0-10 |
| History of persistent asthma | 171 | 5 (2-6) | 0-10 |
| Patient successfully used an AAI before | 171 | 5 (3-7) | 0-10 |
| Patient with mastocytosis | 170 | 5 (3-8) | 0-10 |
| **What would be barriers to prescribe a new device?** |  |  |  |
| I am waiting for more data about this new device | 174 | 8 (7-10) | 0-10 |
| I prefer to prescribe an AAI for which pharmacokinetic-pharmacodynamic data are available | 173 | 7 (5-9) | 0-10 |
| **What measures would help you reduce barriers to a new adrenaline device?** |  |  |  |
| More clinical data | 173 | 9 (7-10) | 1-10 |
| Recommendations from allergy societies | 174 | 9 (7.75-10) | 0-10 |
| Information from the manufacturer | 171 | 7 (5-8) | 0-10 |
| Personal experience | 172 | 7 (5-8) | 0-10 |
| Patient experience | 172 | 7 (5-8) | 0-10 |
| **What impact could new adrenaline devices have at the community level?** |  |  |  |
| Increased awareness of allergy and anaphylaxis in the community | 173 | 7 (5-8) | 1-10 |
| Increased use of adrenaline to treat anaphylaxis in the community at all | 174 | 8 (7-10) | 3-10 |
| More prompt use of adrenaline in patients with severe anaphylaxis | 175 | 9 (7-10) | 0-10 |
| More prompt use of adrenaline in patients with mild to moderate anaphylaxis | 173 | 8 (7-9) | 3-10 |
| Wider access to adrenaline in public spaces | 175 | 8 (6-9) | 0-10 |
| Wider access to adrenaline in schools | 175 | 8 (7-10) | 0-10 |
| Improved global availability of adrenaline | 174 | 8 (6.75-10) | 1-10 |

AAI: adrenaline auto-injector; IQR: interquartile range

Table S2. Comparison of the main characteristics of the participants and responses according to countries. Responses to the adrenaline questionnaire are expressed as median (IQR). Significant p values are in bold.

|  | **France** | **Austria** | **Germany** | **Other countries*** | **p** |
| --- | --- | --- | --- | --- | --- |
| N | 87 | 39 | 20 | 29 |  |
| Median year of practice, y (IQR) | 12 (5-30) | 4 (2-15) | 25.5 (9-30) | 20 (9-30) | **< 0.001** |
| Gender female, n (%) | 67 (77.0) | 26 (66.7) | 12 (60.0) | 21 (72.4) | 0.382 |
| **Medical speciality, n (%)** |  |  |  |  |  |
| Allergology | 62 (71.3) | 0 | 7 (35) | 19 (65.5) | **< 0.001** |
| Paediatric allergology | 9 (10.3) | 3 (7.7) | 2 (10) | 2 (6.9) | **< 0.001** |
| Paediatrics | 9 (10.3) | 34 (87.2) | 2 (10) | 3 (10.3) | **< 0.001** |
| Dermatology | 0 | 2 (5.1) | 9 (45) | 1 (3.4) | **< 0.001** |
| Other specialities | 7 (8) | 0 | 0 | 4 (13.8) | NC |
| **NORA and/or AVN members, n (%)** | 78 (89.7) | 4 (10.3) | 20 (100) | 23 (79.3) |  |
|  |  |  |  |  |  |
| **Questionnaire** |  |  |  |  |  |
| **Use of AAI** |  |  |  |  |  |
| How do you rate the user-friendliness of the AAI? | 9 (7-10) | 8 (6-8) | 8 (8-9.25) | 8 (7-8) | **0.002** |
| How do you rate the user-friendliness of the AAI from the patient perspective? | 6 (5-7) | 6 (4.5-7) | 6.5 (5-8) | 5 (4-7) | 0.401 |
| **What are the main barriers to prescribe an AAI in your practice?** |  |  |  |  |  |
| Uncertainty about the indication or need for an AAI | 3 (1-7) | 4.5 (1-7) | 1 (0-6.75) | 5 (2-6) | 0.314 |
| High cost or lack of insurance coverage | 1 (0-5) | 1 (0-3) | 1 (0-3) | 5 (2-7.5) | **0.001** |
| Concerns about patient adherence (e.g., not carrying or using the AAI correctly) | 6 (3-8) | 5 (2-7) | 5 (3-7) | 6 (5-8) | 0.128 |
| Limited availability or supply issues | 7 (4-9) | 2 (0-3.25) | 5 (1.5-6) | 5 (2-8) | **< 0.001** |
| Patient or caregiver reluctance (e.g., fear of injections, misunderstanding of anaphylaxis severity) | 6 (3-8) | 5 (2.5-7) | 7 (5-8) | 7 (5-8) | 0.282 |
| **What are the most important features you expect from new adrenaline devices to treat anaphylaxis as compared to AAI?** |  |  |  |  |  |
| Detailed pharmacokinetic-pharmacodynamic data | 9 (7-10) | 7 (5-8.25) | 8.5 (7-9.75) | 8 (6.5-9) | 0.011 |
| Optimised dosing with wide dose range (from infancy to elderly, overweight-obese individuals) | 9 (7-10) | 8 (6-9) | 8 (7.25-9) | 8 (7-10) | 0.014 |
| Needle-free device | 8 (6-10) | 8 (7-10) | 8 (5.5-9) | 5 (5.5-9) | 0.14 |
| Availability in public spaces (schools, restaurants, shopping precincts, airports and airplanes, sport stadia…) | 9 (7-10) | 8 (7-10) | 8 (6.75-10) | 8 (6-10) | 0.352 |
| Reduced cost | 7 (5-8) | 7 (4-8) | 8 (5.25-9) | 8 (6.5-9.5) | 0.688 |
| Improved storage conditions adapted to real life conditions | 8.5 (7-10) | 9 (7-9.25) | 8 (7.25-9) | 9 (7-10) | 0.101 |
| Prolonged shelf-life | 9 (8-10) | 9 (7-9.25) | 9 (8-9) | 9 (7-10) | 0.975 |
| Reduced size of the device | 6 (5-8) | 7 (6-9) | 8 (6.25-9) | 7 (5-9) | **0.002** |
| Easy to carry | 8 (6-9) | 9 (8-10) | 9 (8-9) | 9 (6.5-9.5) | 0.033 |
| **A new adrenaline device (by nasal) is available with comparable pharmacokinetic-pharmacodynamic data to AAI and a marketing authorization**  **What would be reasons not to use the new device?** |  |  |  |  |  |
| Overweight patient | 4 (1-5) | 5 (2-6) | 6 (3-7) | 6 (5-8) | **0.001** |
| History of severe anaphylaxis | 5 (2-7) | 7 (5-10) | 7 (3.5-8) | 7 (5-8.5) | **0.001** |
| History of anaphylaxis admitted to intensive care unit | 5 (2-8) | 8 (5-10) | 8 (3.5-8) | 7 (5-9) | **0.001** |
| History of anaphylaxis treated with more than one adrenaline injection | 5.5 (3-8) | 7.5 (5-10) | 7 (3-8.75) | 7 (5-9) | 0.071 |
| History of persistent asthma | 3 (1-5) | 5 (4-7) | 5 (2.5-7.75) | 6 (5-8) | **< 0.001** |
| Patient successfully used an AAI before | 5 (2-7) | 6 (5-8) | 7 (3-8) | 6 (5-8) | 0.012 |
| Patient with mastocytosis | 5 (2-7) | 7 (5-10) | 5 (3.25-9) | 7 (5-9) | **< 0.001** |
| **What would be barriers to prescribe a new device?** |  |  |  |  |  |
| I am waiting for more data about this new device | 9 (7-10) | 8 (5.57-9) | 7.5 (6.25-9) | 8 (6-9.5) | **0.001** |
| I prefer to prescribe an AAI for which pharmacokinetic-pharmacodynamic data are available | 7 (5-9) | 7 (5-9) | 8 (7-9) | 6 (5-8) | 0.131 |
| **What measures would help you reduce barriers to a new adrenaline device?** |  |  |  |  |  |
| More clinical data | 8 (7-10) | 8.5 (7-10) | 9 (8-9.75) | 9 (8-10) | 0.689 |
| Recommendations from allergy societies | 9 (8-10) | 9 (7-10) | 8.5 (7-9) | 8 (7-10) | 0.322 |
| Information from the manufacturer | 7 (5-8) | 9 (7-8) | 7 (5.25-8) | 7 (5-9) | 0.347 |
| Personal experience | 7 (5-8) | 6.5 (5-8) | 8 (7.25-9) | 8 (7-9) | 0.008 |
| Patient experience | 7 (5-8) | 6 (5-8) | 7.5 (5.25-8.75) | 8 (6-9) | 0.184 |
| **What impact could new adrenaline devices have at the community level?** |  |  |  |  |  |
| Increased awareness of allergy and anaphylaxis in the community | 7 (5-8) | 7 (5-8) | 8 (6-8.75) | 8 (6-9) | 0.319 |
| Increased use of adrenaline to treat anaphylaxis in the community at all | 8 (8-10) | 8 (7-9) | 8 (7-9) | 8 (6.5-10) | 0.130 |
| More prompt use of adrenaline in patients with severe anaphylaxis | 9 (8-10) | 8 (7-9) | 9 (7-9.75) | 8 (7-10) | 0.166 |
| More prompt use of adrenaline in patients with mild to moderate anaphylaxis | 8 (7-10) | 8 (7-9) | 8 (7-10) | 8 (7-10) | 0.383 |
| Wider access to adrenaline in public spaces | 8 (7-10) | 7 (5-8) | 7 (4.25-9) | 8 (5.5-10) | 0.036 |
| Wider access to adrenaline in schools | 9 (7-10) | 8 (6-9) | 8 (7-9) | 8 (6.5-10) | 0.52 |
| Improved global availability of adrenaline | 8.5 (7-10) | 7 (5-8) | 9 (6.25-9) | 8 (6.5-10) | 0.015 |

AAI: adrenaline auto-injector; AVN: Allergy-Vigilance Network; IQR: interquartile range; NC: non calculable; NORA: Network for Online Registration of Anaphylaxis

*Other countries: Algeria, Belgium, Bulgaria, Croatia, Italy, Ireland, Luxembourg, Macedonia, Morocco, Netherlands, Poland, Spain, Switzerland, Romania

Table S3. Comparison of the responses to the adrenaline questionnaire according to medical speciality of the participants. Responses to the adrenaline questionnaire are expressed as median (IQR). Significant p values are in bold.

|  | **Allergology** | **Paediatric Allergology** | **Paediatrics** | **Dermatology** | **Other specialities*** | **p** |
| --- | --- | --- | --- | --- | --- | --- |
| N | 88 | 16 | 48 | 12 | 11 |  |
| Median year of practice, y (IQR) | 15  (5.75-30) | 9.5  (4-21) | 10  (3-17) | 12  (5.5-30) | 28.5  (12-33.8) | **0.002** |
| Gender female, n (%) | 67 (53.2) | 10 (7.9) | 34 (27) | 8 (6.3) | 7 (5.6) | 0.727 |
| NORA and/or AVN members,  n (%) | 77 (62.6) | 12 (9.8) | 15 (12.2) | 12 (9.8) | 7 (5.7) | **< 0.001** |
| **Questionnaire** |  |  |  |  |  |  |
| **Use of AAI** |  |  |  |  |  |  |
| How do you rate the user-friendliness of the AAI? | 8 (7-10) | 9 (8-10) | 8 (6-8) | 8 (7-8) | 8 (7.25-8.75) | 0.006 |
| How do you rate the user-friendliness of the AAI from the patient perspective? | 6 (4.75-7) | 7 (5-8.25) | 5 (4-7) | 6 (5-7.5) | 7 (5-8.5) | 0.084 |
| **What are the main barriers to prescribe an AAI in your practice?** |  |  |  |  |  |  |
| Uncertainty about the indication or need for an AAI | 3 (1-7) | 0 (0-6.5) | 2 (1-7) | 2 (1-6) | 4.5 (2.25-6.75) | 0.461 |
| High cost or lack of insurance coverage | 1 (0-5) | 1 (0-6.25) | 1 (0-3) | 3 (0.5-4.5) | 3 (1.25-4.5) | 0.273 |
| Concerns about patient adherence (e.g., not carrying or using the AAI correctly) | 6 (3-7.25) | 5 (2-8) | 5 (3-7) | 5 (1.5-8) | 7.5 (5-9) | 0.089 |
| Limited availability or supply issues | 5 (2-8) | 5 (2-8) | 2 (1-5) | 5 (1.5-8) | 7 (4.25-7.75) | **< 0.001** |
| Patient or caregiver reluctance (e.g., fear of injections, misunderstanding of anaphylaxis severity) | 6 (3-8) | 5 (2-9) | 5 (4-7) | 6 (5-8.5) | 6.5 (6-7.75) | 0.621 |
| **What are the most important features you expect from new adrenaline devices to treat anaphylaxis as compared to AAI?** |  |  |  |  |  |  |
| Detailed pharmacokinetic-pharmacodynamic data | 9 (7-10) | 9 (6.75-10) | 8 (6-9) | 8 (7-8.5) | 8 (7.25-9.75) | 0.013 |
| Optimised dosing with wide dose range (from infancy to elderly, overweight-obese individuals) | 9 (7-10) | 8.5 (7.25-10) | 8 (7-8) | 9 (8-9) | 8 (7.25-9.75) | 0.006 |
| Needle-free device | 8 (6.75-9) | 9.5 (6.5-10) | 8 (7-10) | 9 (7.5-10) | 6.5 (5.25-8) | 0.074 |
| Availability in public spaces (schools, restaurants, shopping precincts, airports and airplanes, sport stadia…) | 8 (6-10) | 6 (4.75-9) | 8 (7-9) | 9 (7.5-10) | 7 (5.25-9) | 0.403 |
| Reduced cost | 7 (5-8) | 8 (4-9) | 7 (5-8) | 9 (7.5-9) | 7 (4.25-8) | 0.166 |
| Improved storage conditions adapted to real life conditions | 8.5 (7-10) | 7.5 (4.75-10) | 8 (7-10) | 9 (8-10) | 8 (6-9.75) | 0.464 |
| Prolonged shelf-life | 9 (8-10) | 9 (7-10) | 9 (7-10) | 9 (8-10) | 8.5 (8-9.75) | 0.933 |
| Reduced size of the device | 7 (5-8.25) | 6 (4.75-9) | 7 (6-9) | 9 (7.5-9.5) | 6 (5-7) | 0.017 |
| Easy to carry | 8 (6.75-9) | 7.5 (5.75-9.25) | 8 (8-9) | 9 (8-10) | 7 (7-9.5) | 0.185 |
| **A new adrenaline device (by nasal) is available with comparable pharmacokinetic-pharmacodynamic data to AAI and a marketing authorization**  **What would be reasons not to use the new device?** |  |  |  |  |  |  |
| Overweight patient | 5 (2-7) | 4 (0-5) | 5 (3-7) | 6 (2-9.5) | 5 (1.5-6) | 0.241 |
| History of severe anaphylaxis | 6 (2-8) | 5.5 (1-8) | 7 (5-8) | 7 (2.5-9.5) | 5 (3.25-7.75) | 0.294 |
| History of anaphylaxis admitted to intensive care unit | 6 (3-8) | 5 (1-5.5) | 8 (5-10) | 8 (2.5-9.5) | 5 (5-8) | 0.071 |
| History of anaphylaxis treated with more than one adrenaline injection | 7 (4-9) | 5.5 (1.75-8) | 7 (4-10) | 8 (2.5-9) | 7 (5-8) | 0.352 |
| History of persistent asthma | 3 (1-6) | 5.5 (1.75-8) | 7 (4-10) | 5 (3-8.5) | 5 (3.5-6) | 0.083 |
| Patient successfully used an AAI before | 5 (2.75-7) | 5.5 (4.25-8.25) | 5 (5-7) | 7 (5-9) | 7 (5.5-9) | 0.037 |
| Patient with mastocytosis | 5 (2-7) | 5 (1-5.5) | 6 (3-8) | 8 (3-9) | 6 (4.25-9) | 0.157 |
| **What would be barriers to prescribe a new device?** |  |  |  |  |  |  |
| I am waiting for more data about this new device | 8 (6-10) | 9.5 (7.75-10) | 8 (6-9) | 7 (5.5-9.5) | 9.5 (8-10) | 0.317 |
| I prefer to prescribe an AAI for which pharmacokinetic-pharmacodynamic data are available | 7 (5-8) | 7.5 (3.5-9.25) | 8 (6-9) | 7 (6.5-9) | 7 (5-8.5) | 0.472 |
| **What measures would help you reduce barriers to a new adrenaline device?** |  |  |  |  |  |  |
| More clinical data | 9 (7-10) | 10 (7.75-10) | 8 (7-10) | 9 (8-9.5) | 8.5 (8-10) | 0.370 |
| Recommendations from allergy societies | 9 (7-10) | 9 (5.75-10) | 8 (7-10) | 9 (8-9.5) | 8.5 (8-10) | 0.900 |
| Information from the manufacturer | 7 (5.75-8) | 6.5 (5-8.25) | 6 (5-8) | 8 (6.5-9.5) | 7 (5.25-8.75 | 0.422 |
| Personal experience | 7 (5-8) | 7.5 (5-10) | 7 (5-8) | 9 (8-9.5) | 6.5 (5.25-9.5) | 0.062 |
| Patient experience | 7 (5-8.25) | 7.5 (5-8.25) | 7 (5-8) | 8 (6.5-9) | 6 (5-8.25) | 0.616 |
| **What impact could new adrenaline devices have at the community level?** |  |  |  |  |  |  |
| Increased awareness of allergy and anaphylaxis in the community | 7 (5-8.25) | 6.5 (5-9) | 7 (5-8) | 8 (5.5-9.5) | 5.5 (5-7.75) | 0.373 |
| Increased use of adrenaline to treat anaphylaxis in the community at all | 8 (7-9) | 9.5 (8.75-10) | 8 (7-9) | 8 (7-9.5) | 7 (5.25-8.75) | 0.057 |
| More prompt use of adrenaline in patients with severe anaphylaxis | 9 (8-9.25) | 6 (4.75-9) | 9 (7-10) | 9 (7-9.5) | 7 (5-9.75) | 0.042 |
| More prompt use of adrenaline in patients with mild to moderate anaphylaxis | 8.5 (8-10) | 9 (7.5-10) | 7 8 (7-9) | 7 (6.5-9.5) | 7.5 (7-9.5) | 0.109 |
| Wider access to adrenaline in public spaces | 8 (6-9) | 9 (5-10) | 7 (5-8) | 8 (5-9.5) | 8 (5-10) | 0.186 |
| Wider access to adrenaline in schools | 9 (7-10) | 9 (6.75-10) | 8 (7-9) | 8 (6.5-9.5) | 8 (6.25-9.75) | 0.255 |
| Improved global availability of adrenaline | 9 (7-10) | 9.5 (6.5-10) | 8 (5-9) | 9 (6.5-10) | 6.5 (5-8.75) | 0.046 |

AAI: adrenaline auto-injector; IQR: interquartile range; AVN: Allergy-Vigilance Network; IQR: interquartile range; NORA: Network for Online Registration of Anaphylaxis

*Other medical specialities: pneumology (n=13), internal medicine (n=5), otorhinolaryngology (n=1)

Figure S1. Expectations from new adrenaline devices to treat anaphylaxis compared to adrenaline auto-injectors.

AAI: adrenaline auto-injector; PK-PD: pharmacokinetic-pharmacodynamic

The results are presented in bars for each item with the distribution according to the percentage of responses ranked from “not important” (0-3), “neutral” (4-7), “very important” (8-10).

Figure S2. Barriers to use a new nasal adrenaline device.

AAI: adrenaline auto-injector; PK-PD: pharmacokinetic-pharmacodynamic; ICU: intensive care unit

The results are presented in bars for each item with the distribution according to the percentage of responses ranked from “not important” (0-3), “neutral” (4-7), “very important” (8-10). Figure S3. Measures useful to reduce barriers to a new adrenaline device.

The results are presented in bars for each item with the distribution according to the percentage of responses ranked from “not important” (0-3), “neutral” (4-7), “very important” (8-10).

Figure S4. Impact expected from new adrenaline devices at the community level.

The results are presented in bars for each item with the distribution according to the percentage of responses ranked from “not important” (0-3), “neutral” (4-7), “very important” (8-10).

**Alternatives to injectable adrenaline for anaphylaxis – An Anaphylaxis Registry (NORA) and Allergy-Vigilance Network (AVN) survey**

The objective of this electronic survey is to assess limitations and needs regarding adrenaline auto-injector (AAI) use but also expectations about alternative forms of adrenaline. This survey has been approved by the NORA scientific committee and is presented to participants of the 8^th^ International conference of the NORA. The questionnaire is anonymous and the results will be analysed and submitted for publication.

*Indicating the question type*

- *Radio button (single selection only)*
- *Multiple choice (multiple selections allowed)*

**General information**

1. **In which country do you practice?**

<free text>

1. **For how many years are you practicing in allergology?**

<free text>

1. **Please indicate your gender**

- Female
- Male
- Intersexual/ diverse
- Prefer not to say

1. **Please indicate your medical speciality**

- Allergology (incl. paediatric allergology)
- Pediatrics
- Dermatology
- Internal medicine
- Pneumology
- Otorhinolaryngology (ENT)

1. **Are you an active member of the Anaphylaxis Registry (NORA) or the Allergy-Vigilance Network (AVN)?**

- Yes
- No

**Adrenaline Auto-injector (AAI)**

1. **Have you ever used yourself an AAI to treat a patient suffering from anaphylaxis?**

- Yes
- No

*If Yes,*

- 1. **How often did you use an AAI to treat anaphylaxis?**

<10 times

10-50 times

51-100 times

>100 times

Don’t know/ Can’t remember

- 1. **How do you rate the user-friendliness of the AAI?**

Very difficult to use
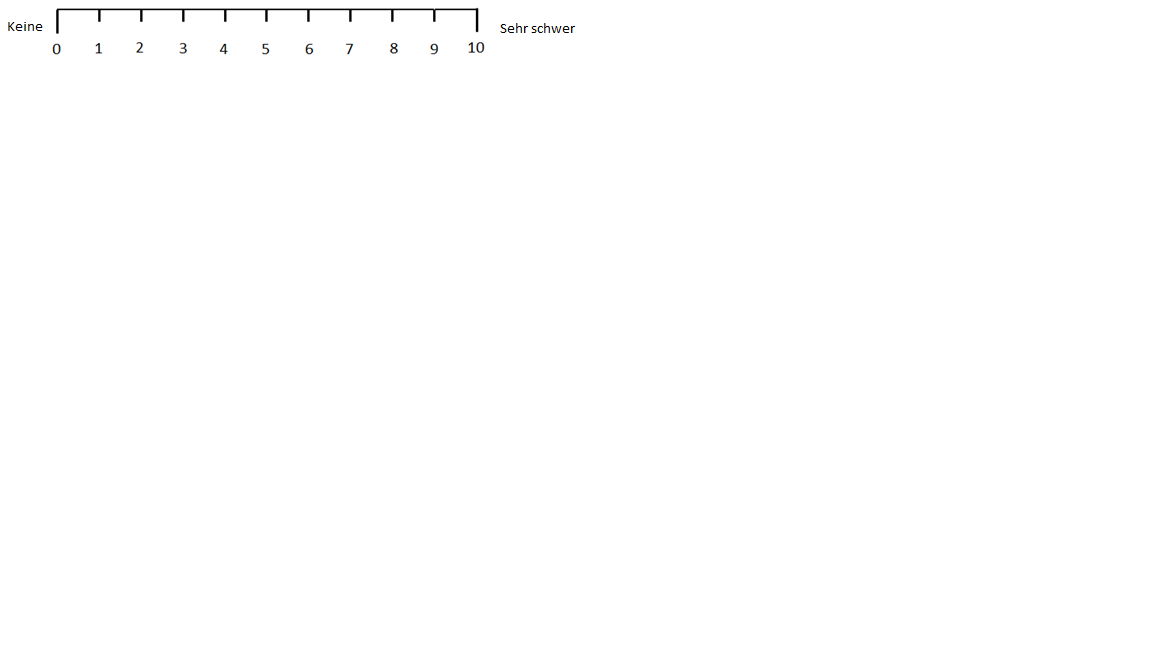
 Very easy to use

**6.3 How do you rate the user-friendliness of the AAI from the patient perspective?**

Very difficult to use
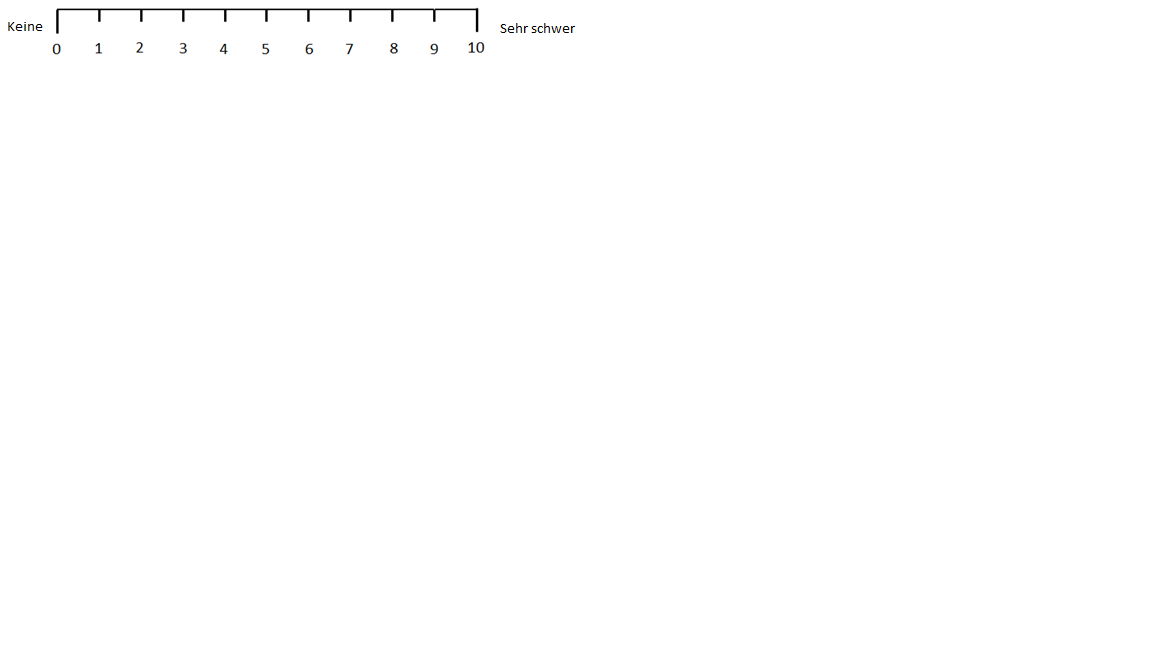
 Very easy to use

1. **What are the main barriers to prescribe an AAI in your practice?**
   Please rank the answers from the list below (0 Not at all important - 10 Very important)

**Uncertainty about the indication or need for an AAI**Not at all important
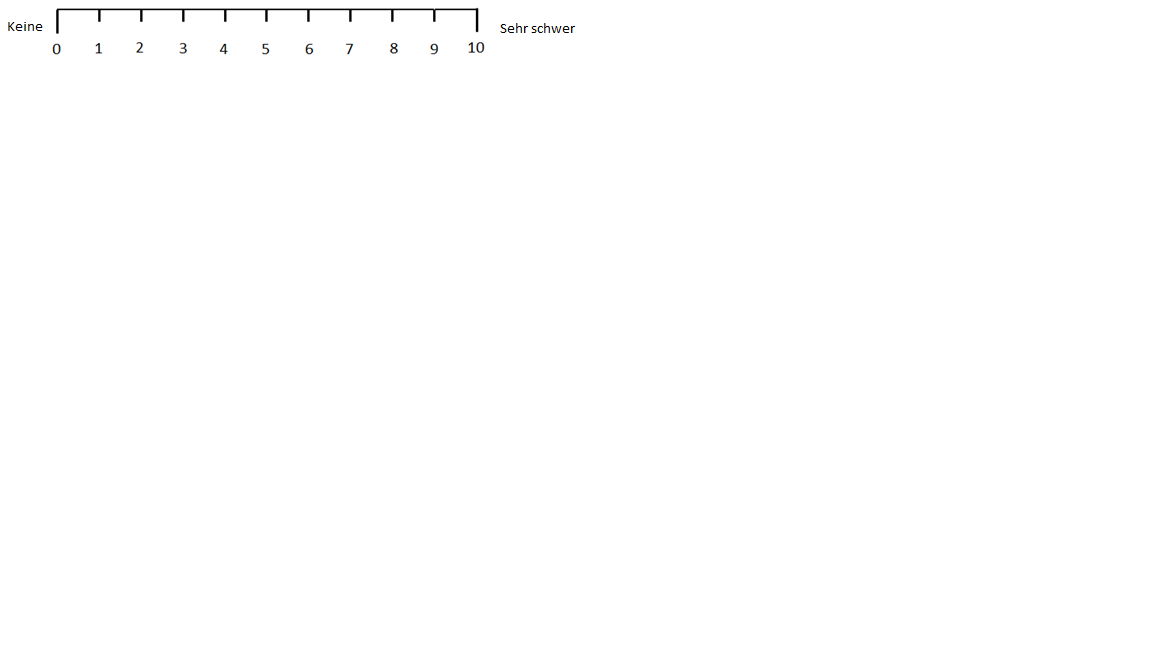
 Very important

**High cost or lack of insurance coverage**Not at all important
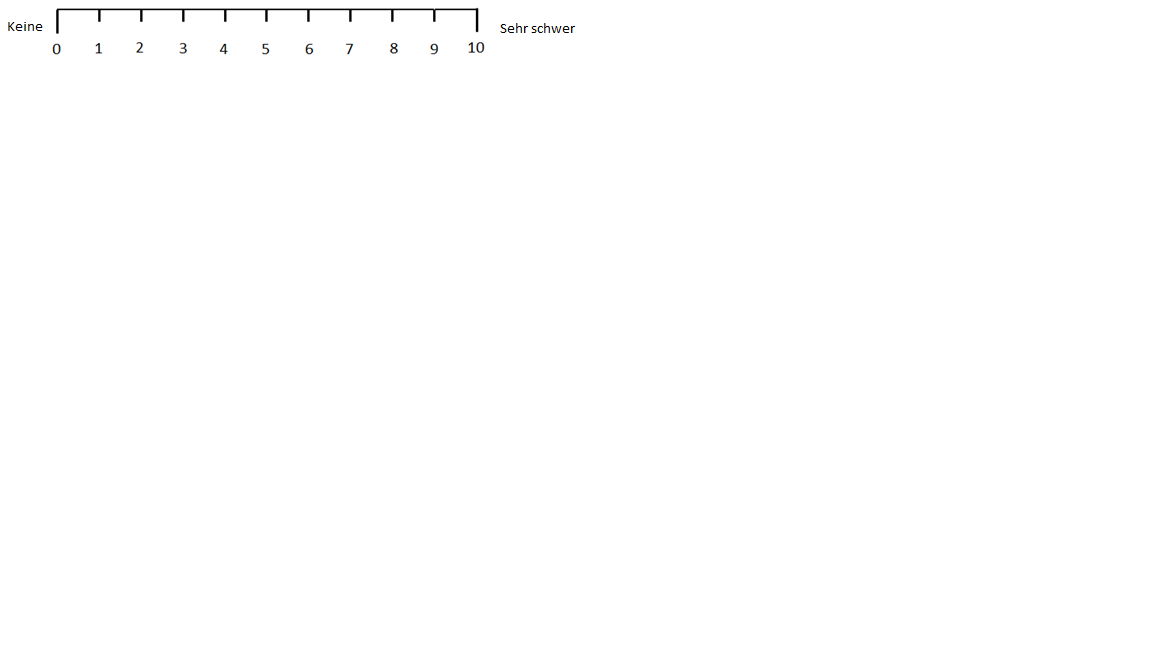
 Very important

**Concerns about patient adherence (e.g., not carrying or using the AAI correctly)**Not at all important
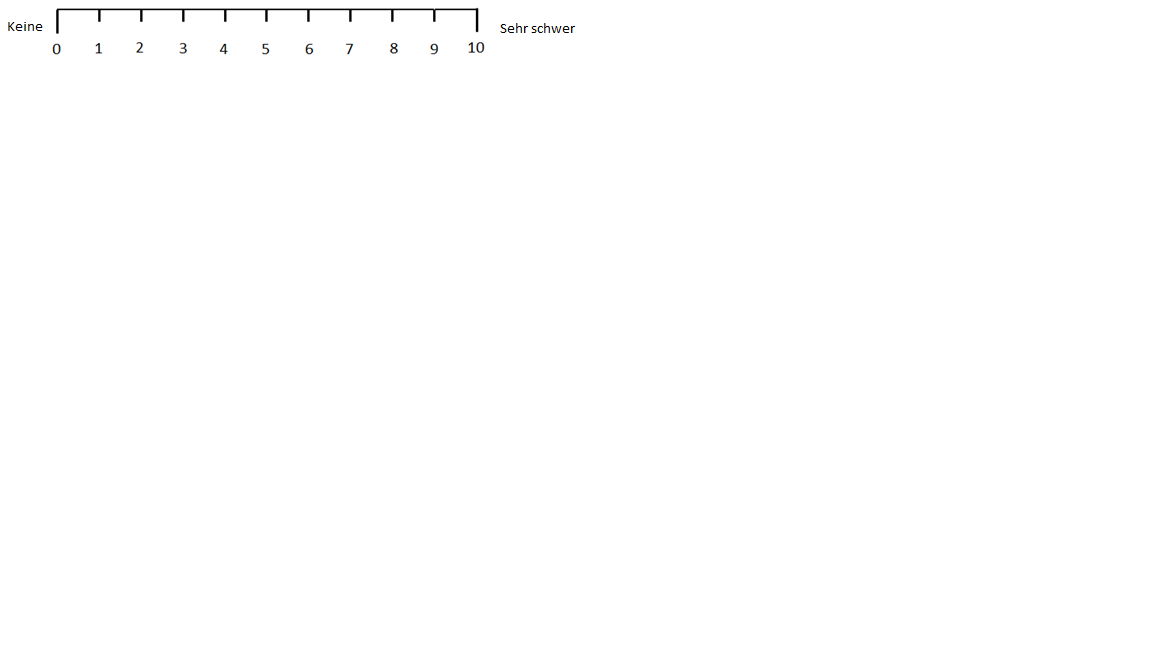
 Very important

**Limited availability or supply issues**
Not at all important
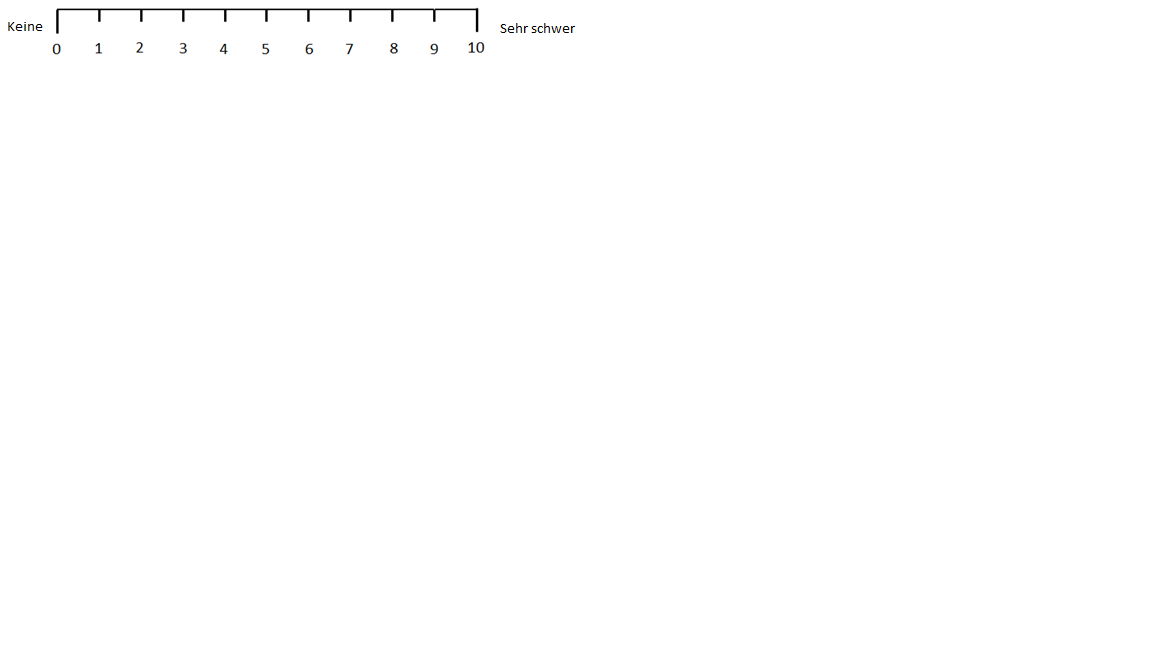
 Very important

**Patient or caregiver reluctance (e.g., fear of injections, misunderstanding of anaphylaxis severity)**
Not at all important
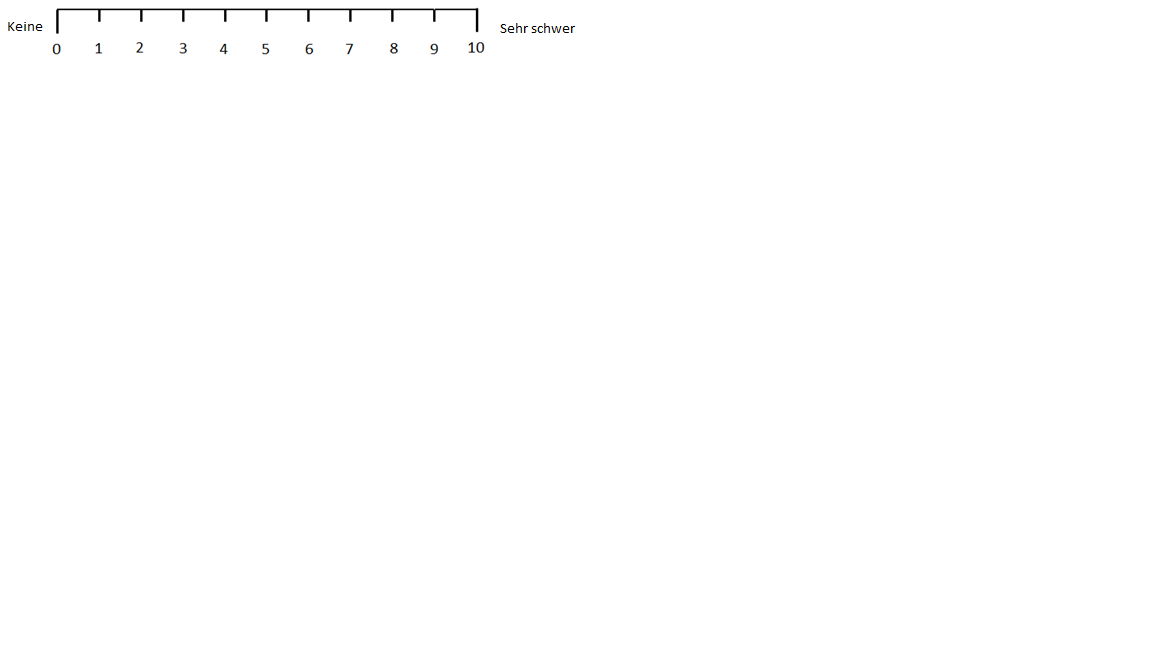
 Very important

**Other (please specify): ___________**Not at all important
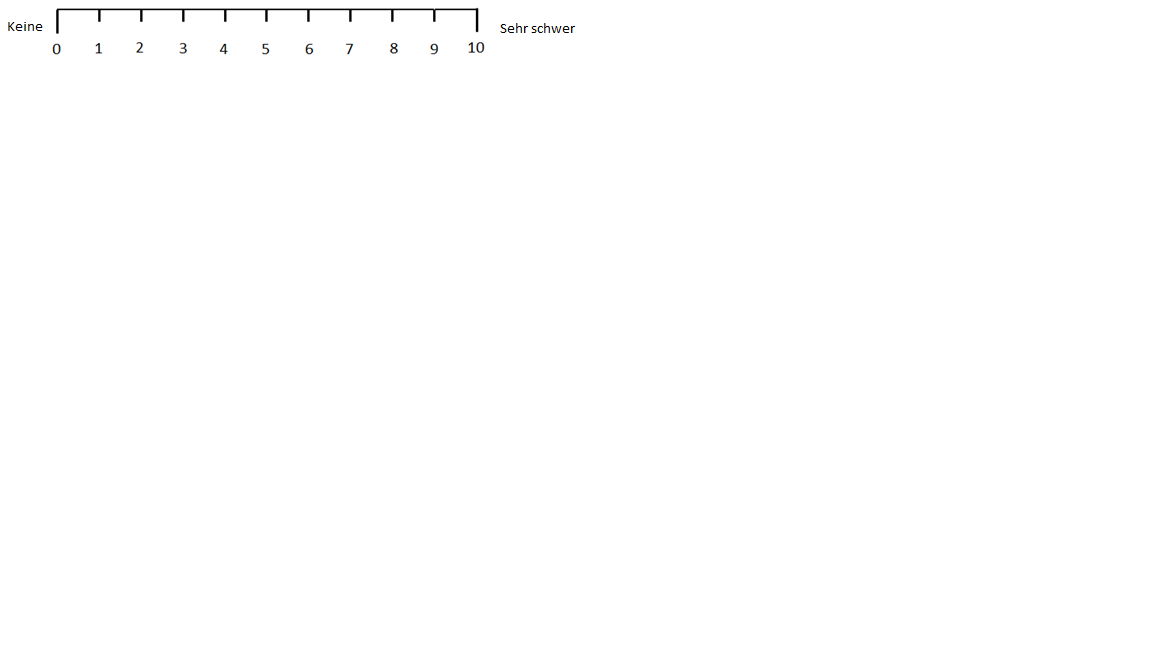
 Very important

**New routes of adrenaline administration and devices are being studied and on clinical validation**

1. **What are the most important features you expect from new adrenaline devices to treat anaphylaxis as compared to AAI?**
   *Please rank the answers from the list below (0* Not at all important *- 10* Very important*)*

**Detailed pharmacokinetic-pharmacodynamic data**
Not at all important
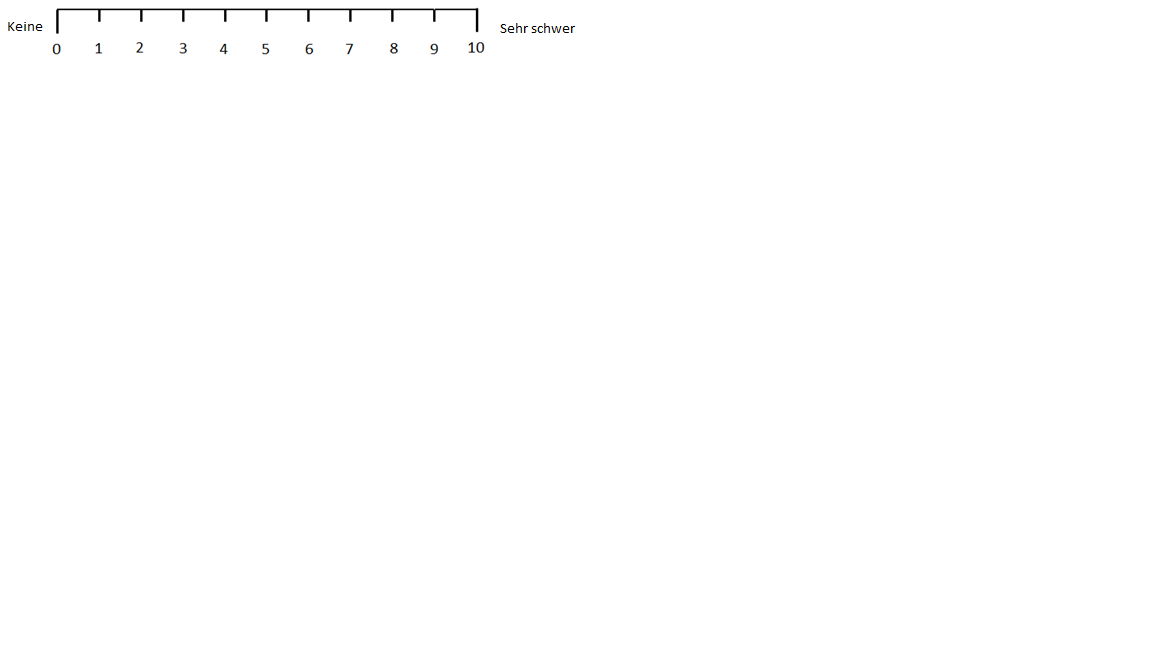
 Very important

**Optimised dosing with wide dose range (from infancy to elderly, overweight-obese individuals)**
Not at all important
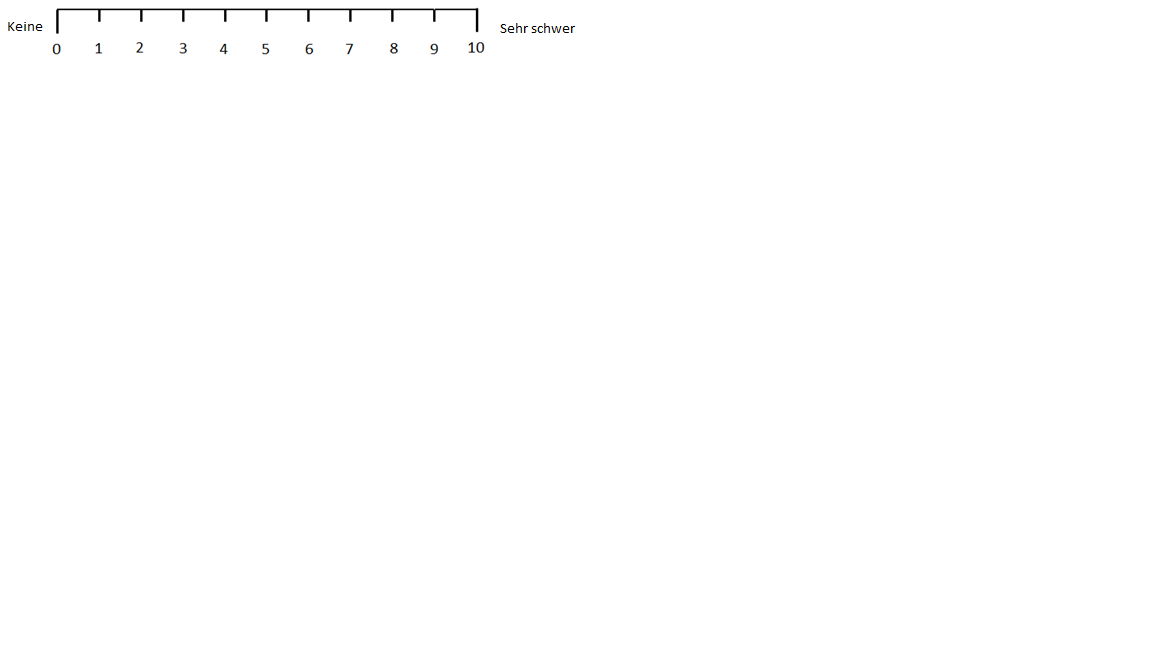
 Very important

**Needle-free device**
Not at all important
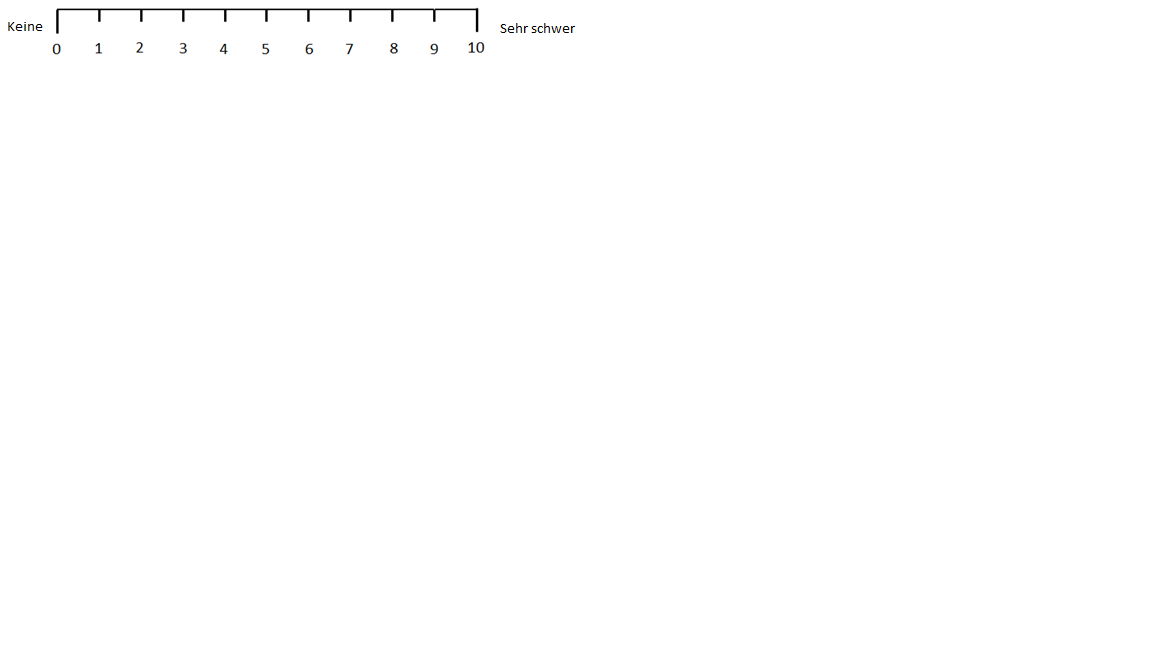
 Very important

**Availability in public spaces (schools, restaurants, shopping precincts, airports and airplanes, sport stadia…)**
Not at all important
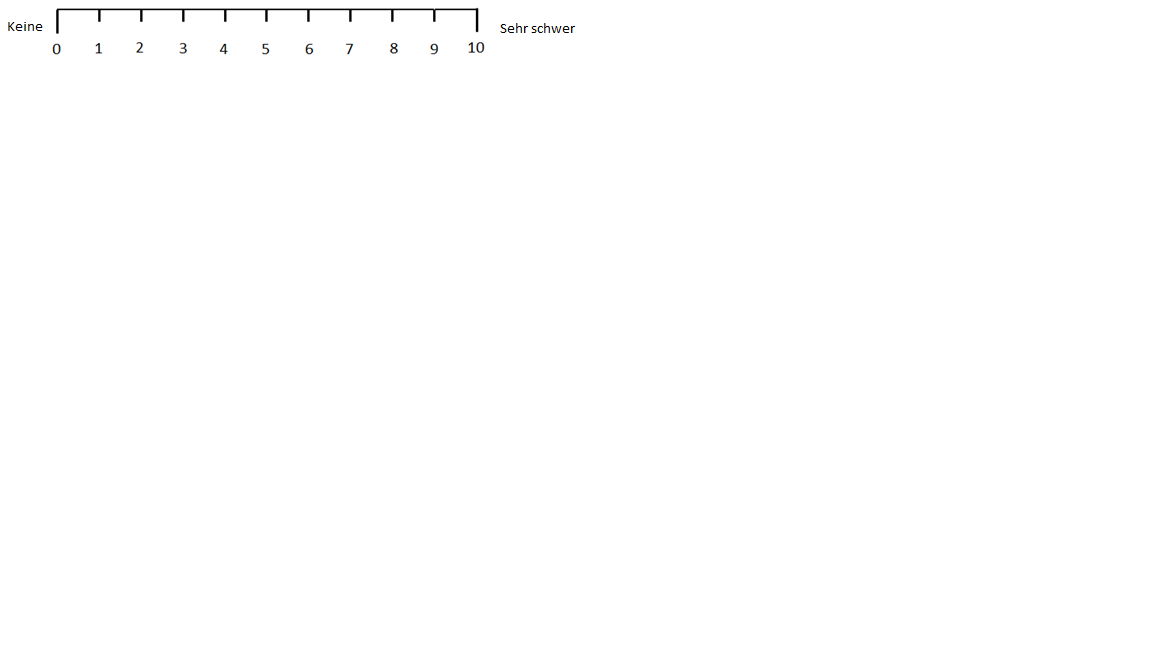
 Very important

**Reduced cost**
Not at all important
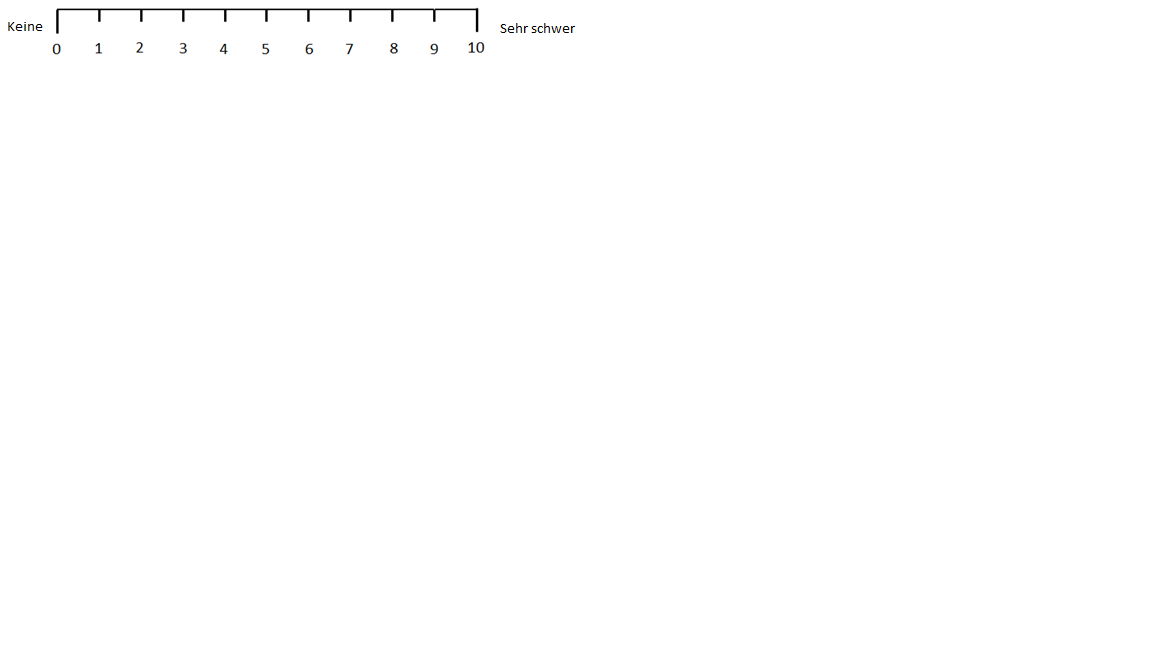
 Very important

**Improved storage conditions adapted to real life conditions**
Not at all important
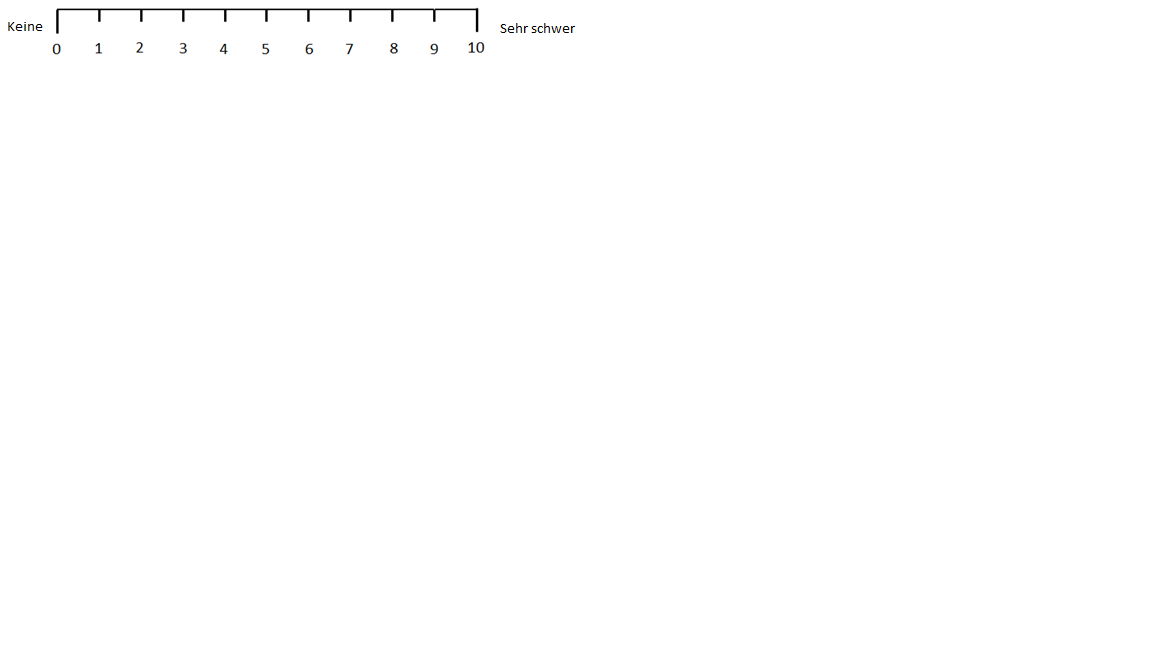
 Very important

**Prolonged shelf-life**
Not at all important
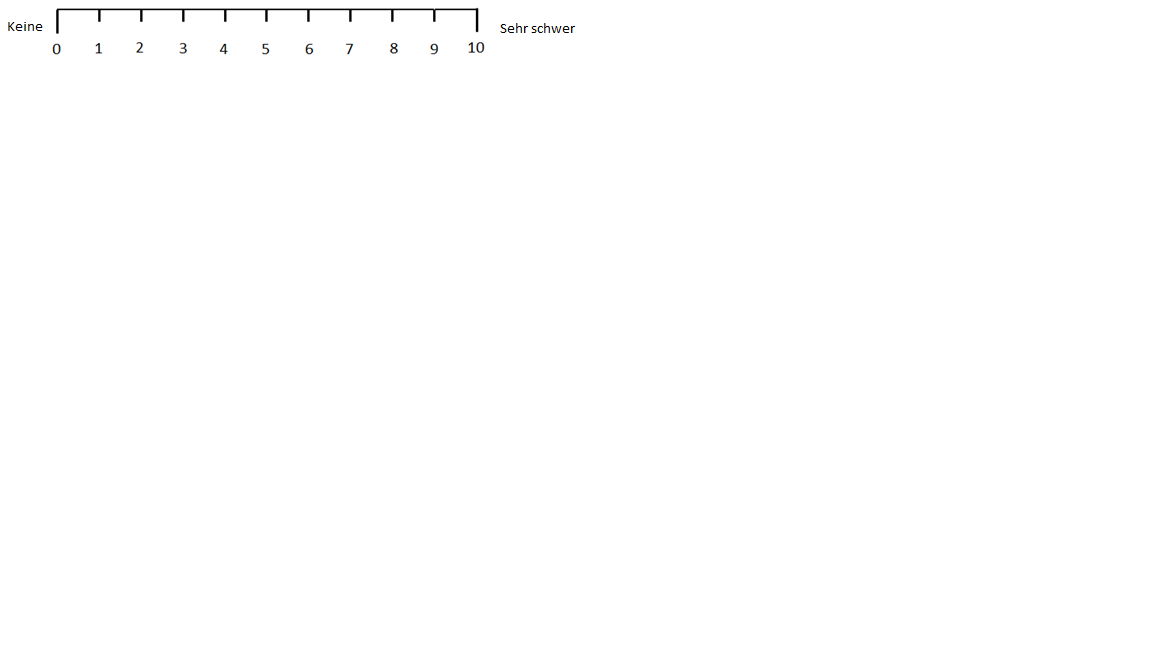
 Very important

**Reduced size of the device**
Not at all important
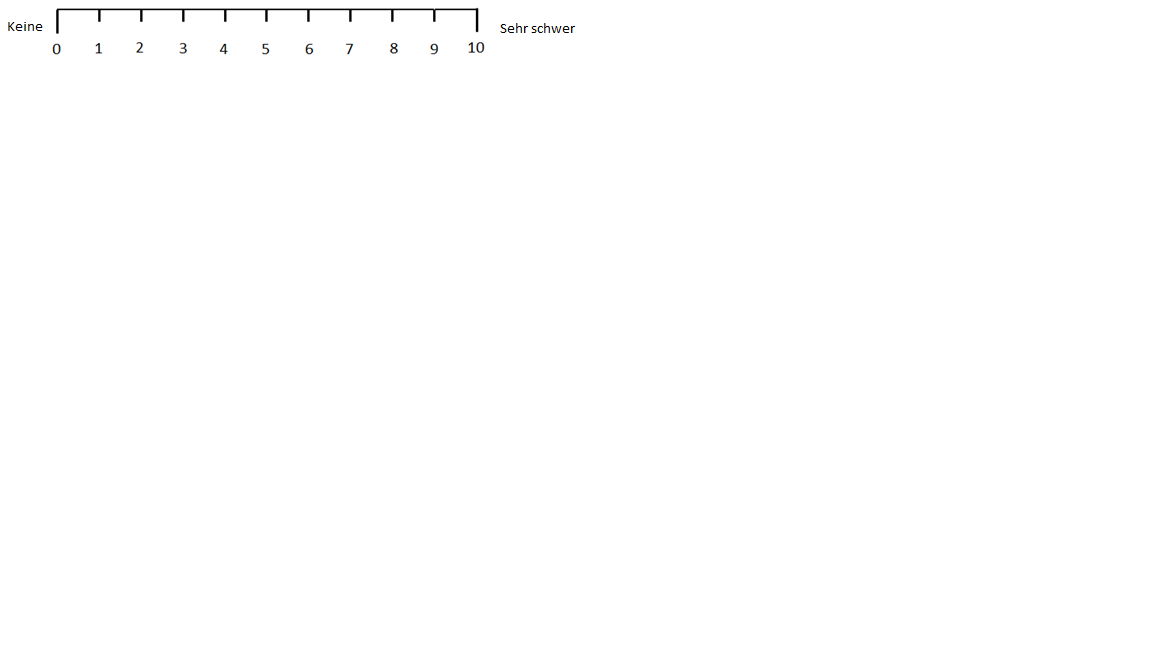
 Very important

**Easy to carry**
Not at all important
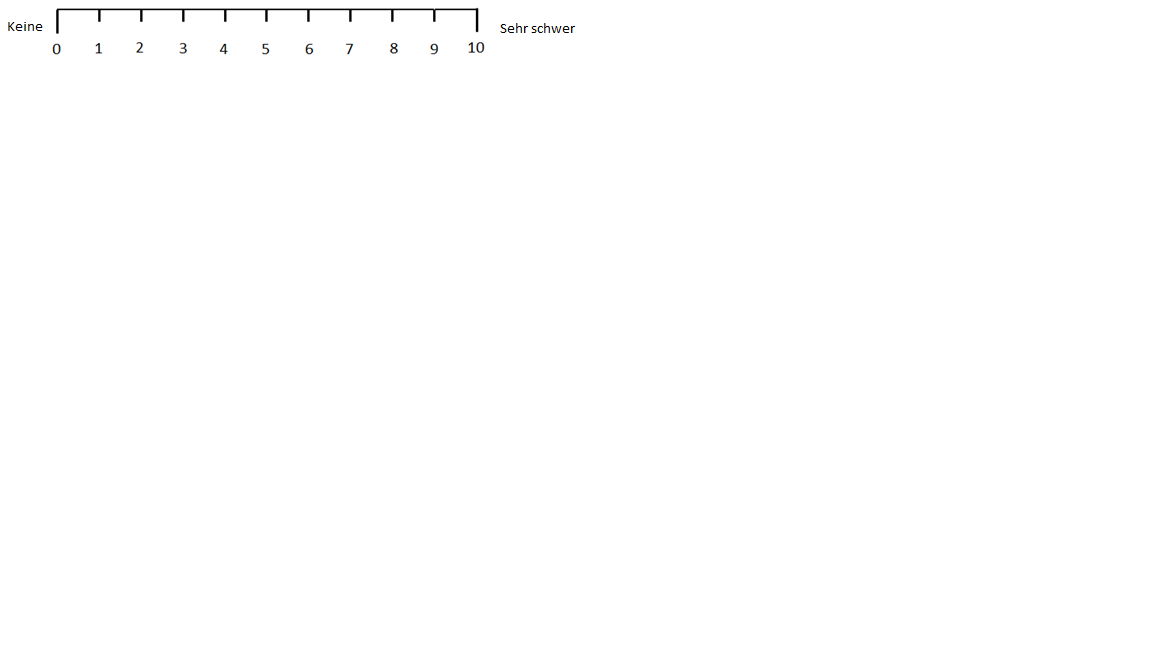
 Very important

**A new adrenaline device (by nasal) is available with comparable pharmacokinetic-pharmacodynamic data to AAI and a marketing authorization**

1. **What would be reasons not to use the new device?**
   *Please rank the answers from the list below (0 Strongly disagree - 10 Strongly agree)*

**Overweight patient**
Strongly disagree
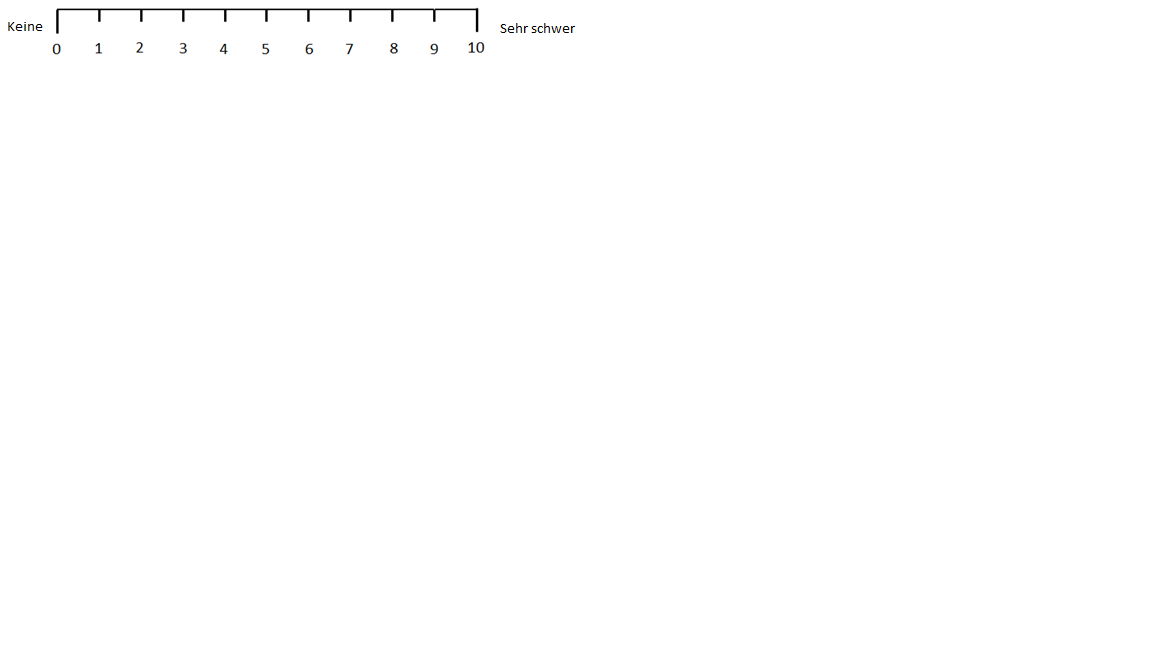
 Strongly agree

**History of severe anaphylaxis**
Strongly disagree
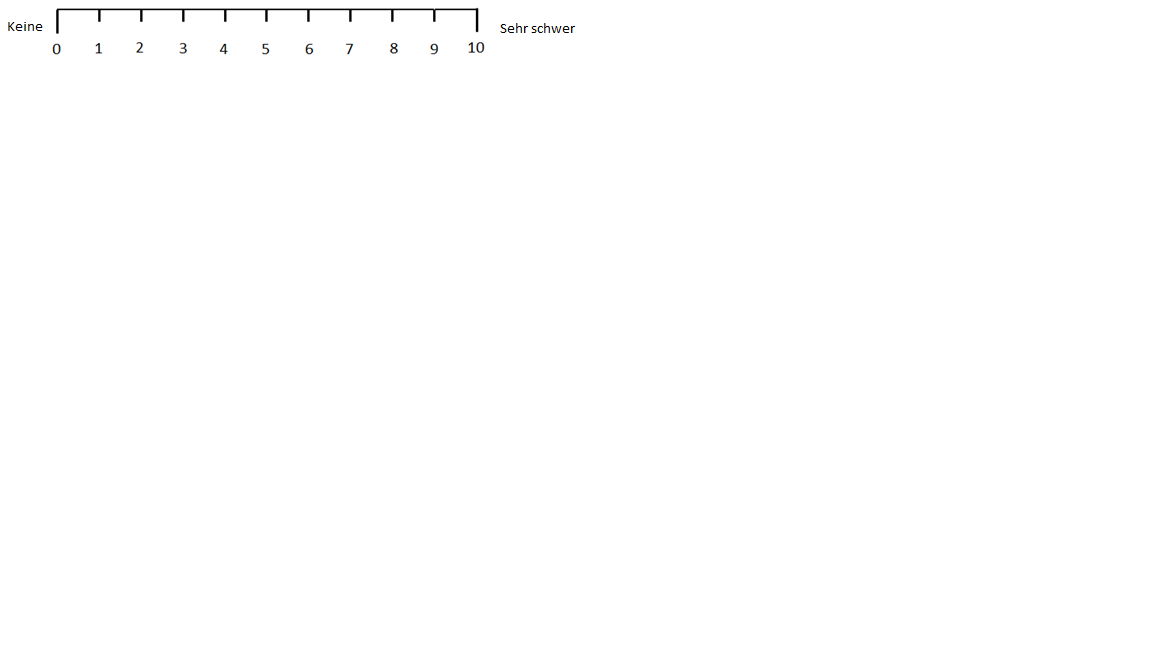
 Strongly agree

**History of anaphylaxis admitted to intensive care unit**
Strongly disagree
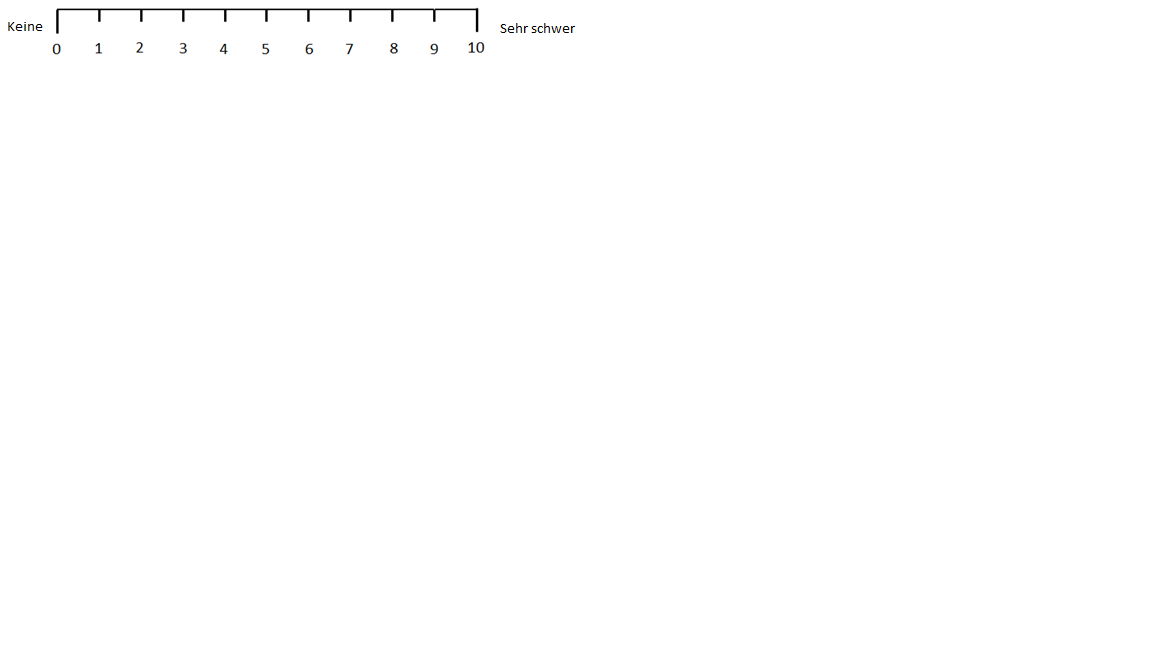
 Strongly agree

**History of anaphylaxis treated with more than one adrenaline injection**
Strongly disagree
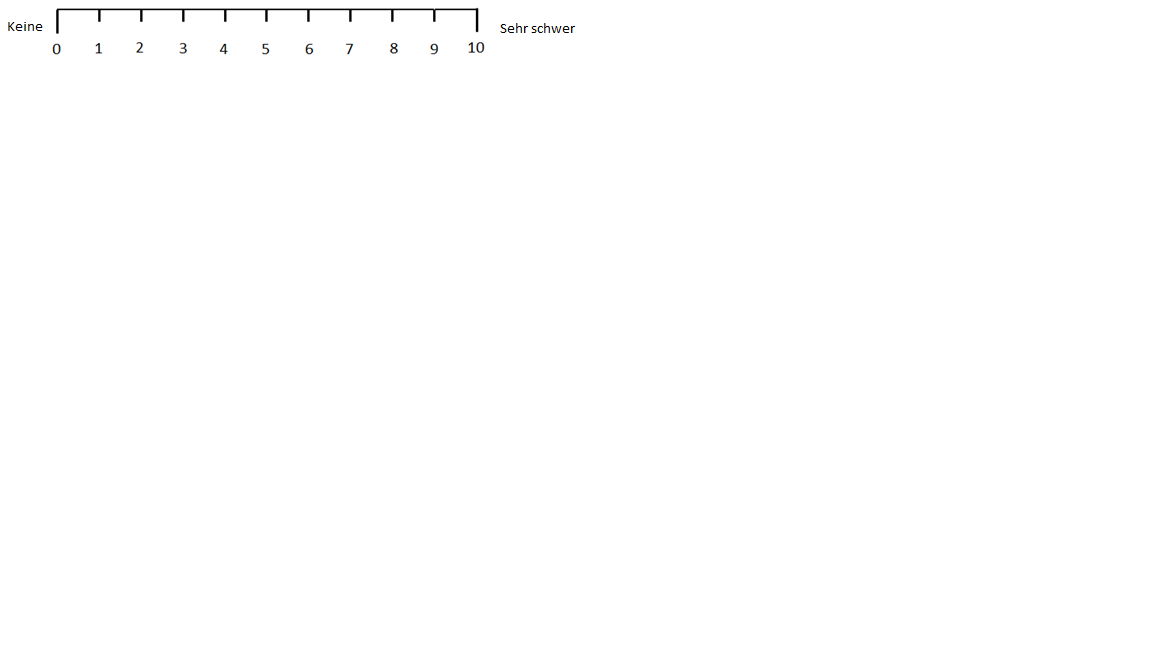
 Strongly agree

**History of persistent asthma**
Strongly disagree
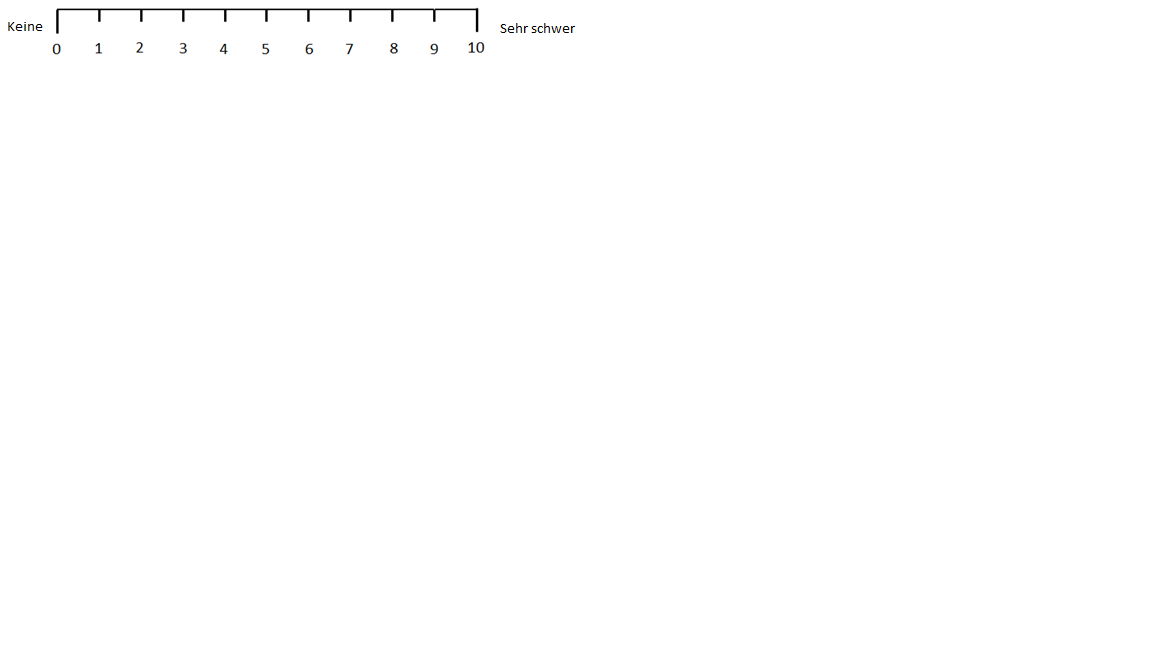
 Strongly agree

**Patient successfully used an AAI before**
Strongly disagree
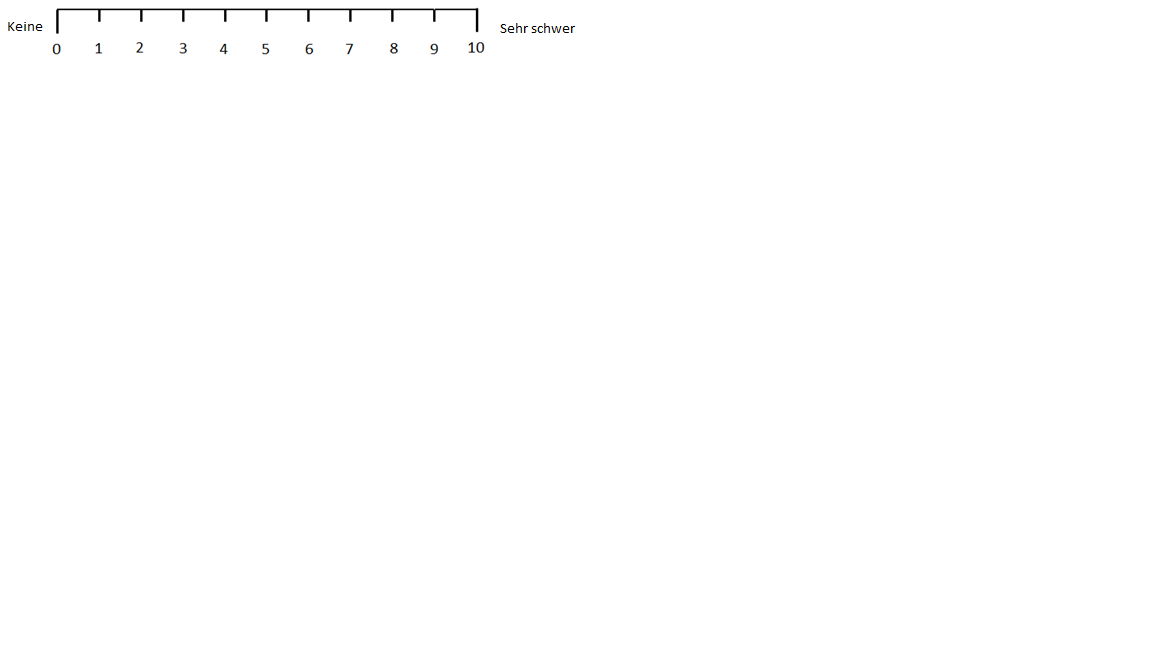
 Strongly agree

**Patient with mastocytosis**
Strongly disagree
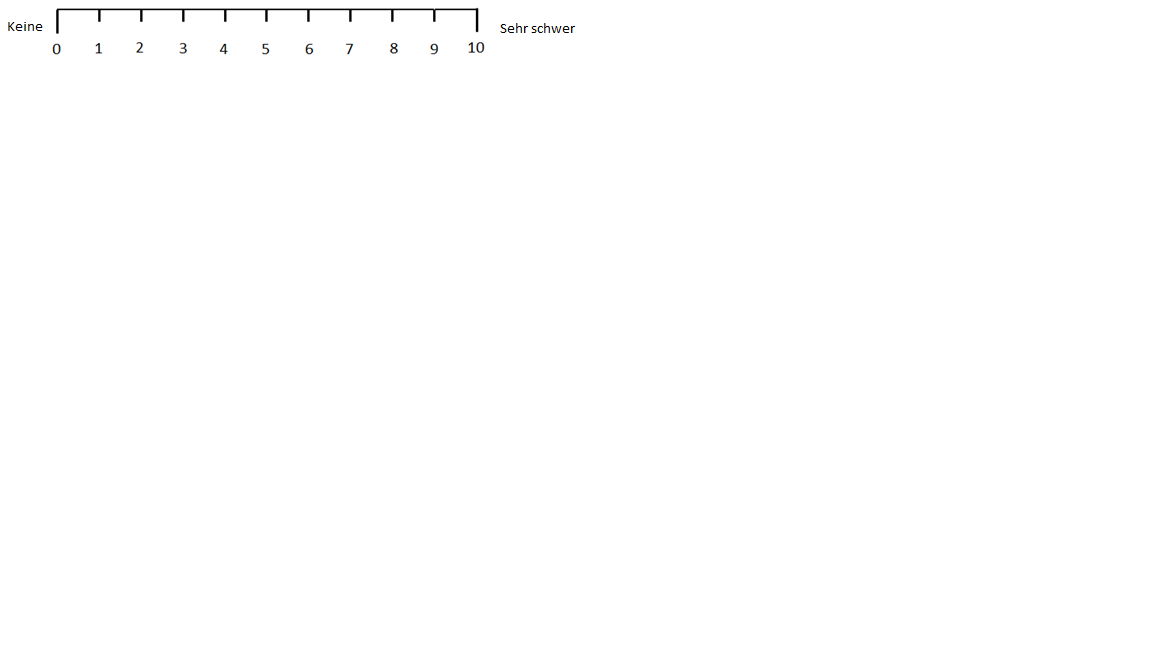
 Strongly agree

**Other indication to prescribe AAI (please specify): ___________**
Strongly disagree
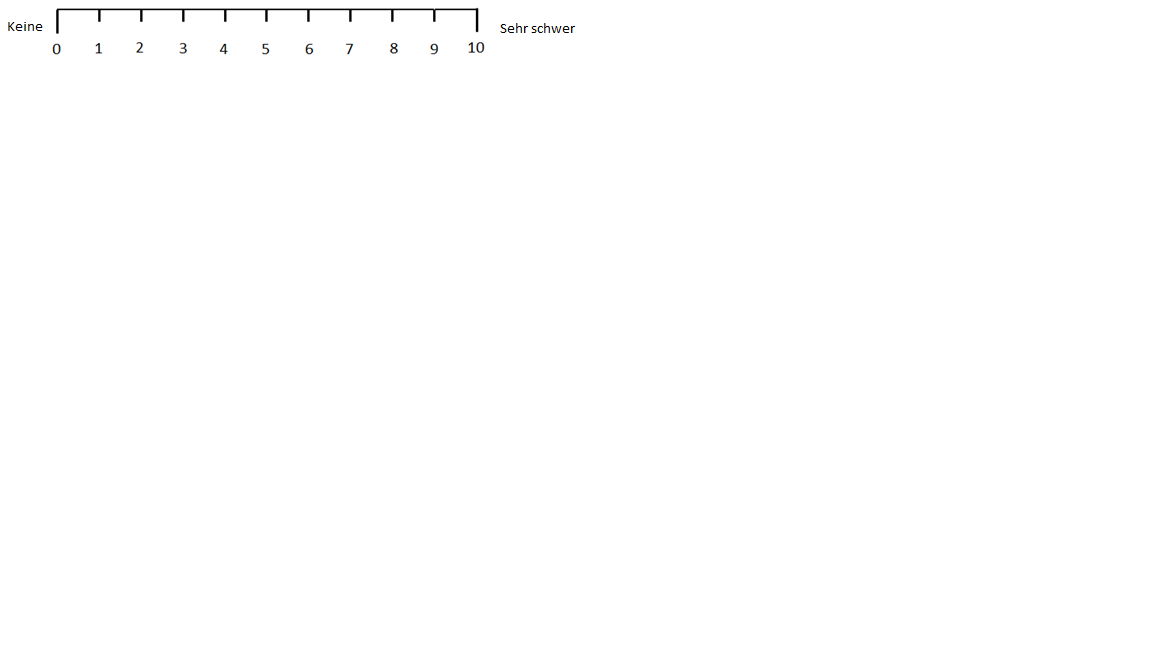
 Strongly agree

1. **What would be barriers to prescribe a new device?***Please rank the answers from the list below (0 Strongly disagree - 10 Strongly agree)*

**I am waiting for more data about this new device**
Strongly disagree
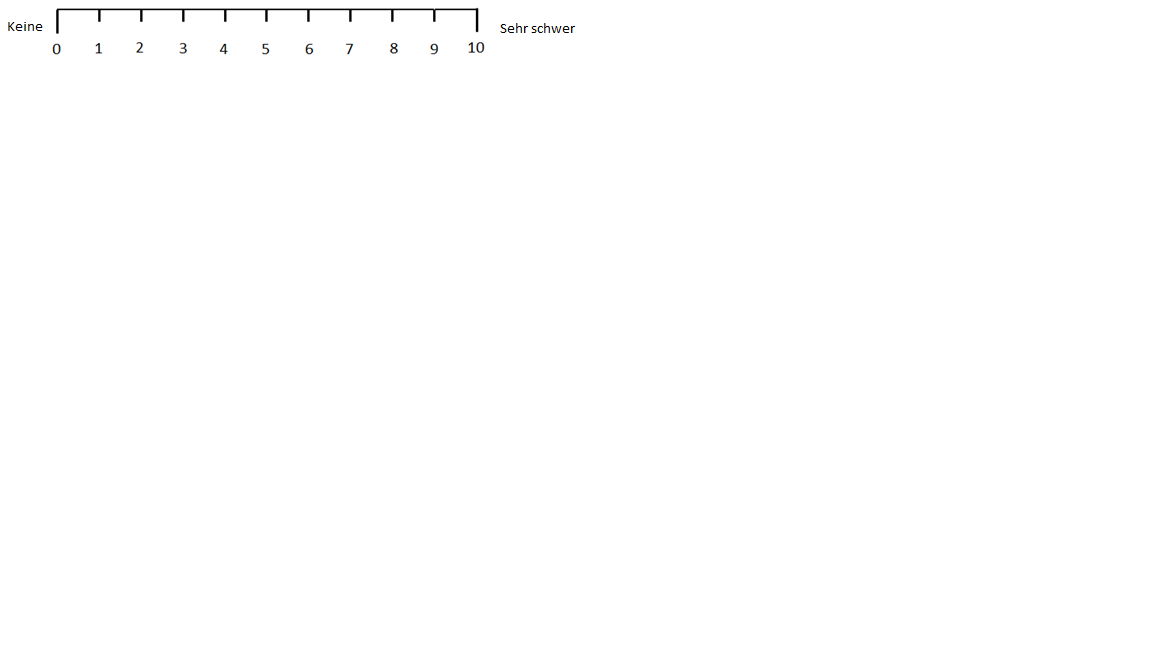
 Strongly agree

**I prefer to prescribe an AAI for which pharmacokinetic-pharmacodynamic data are available**
Strongly disagree
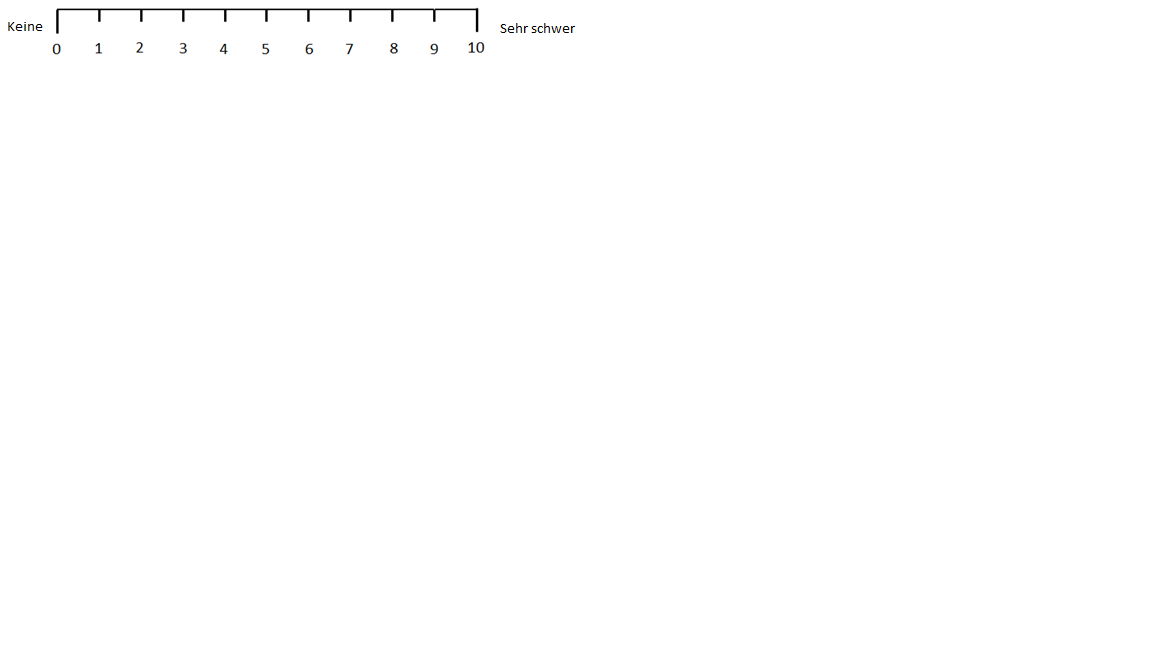
 Strongly agree

**Other response (please specify): ___________**
Strongly disagree
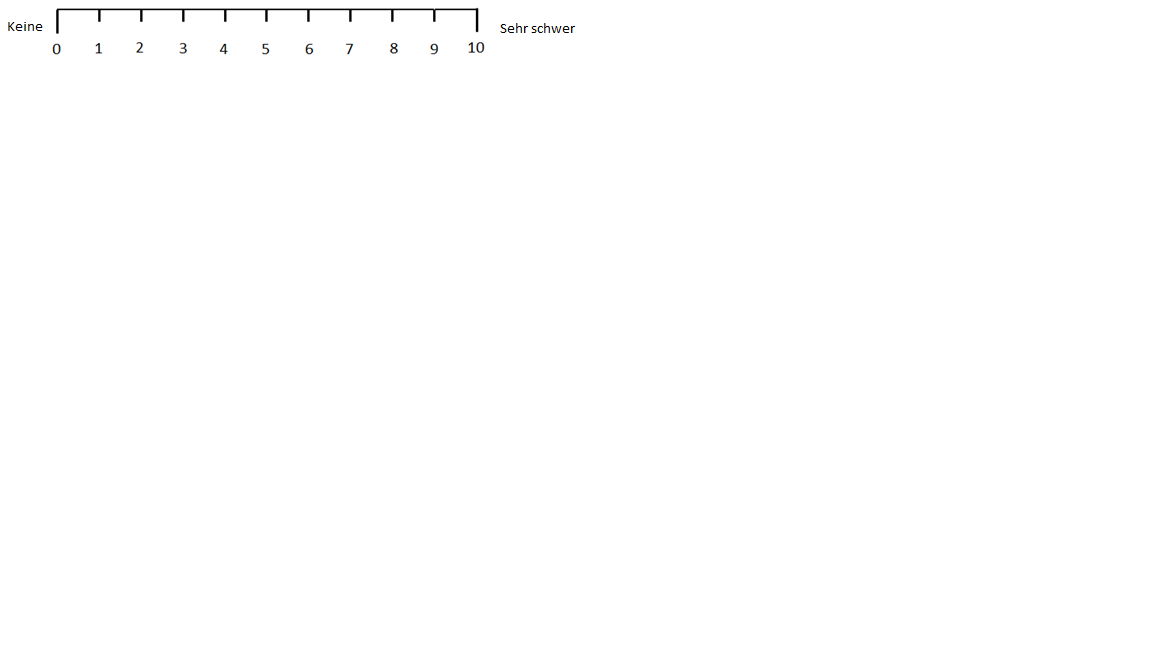
 Strongly agree

1. **What measures would help you reduce barriers to a new adrenaline device?***Please rank the answers from the list below (0 Strongly disagree - 10 Strongly agree)*

**More clinical data**
Not at all important
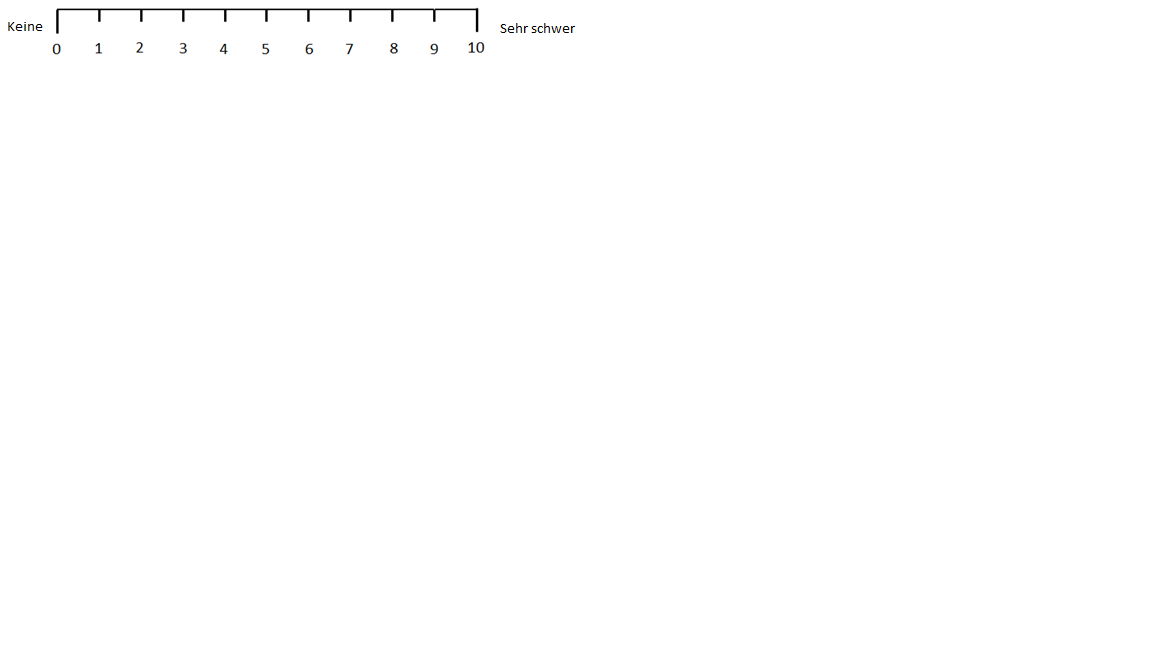
 Very important

**Recommendations from allergy societies**
Not at all important
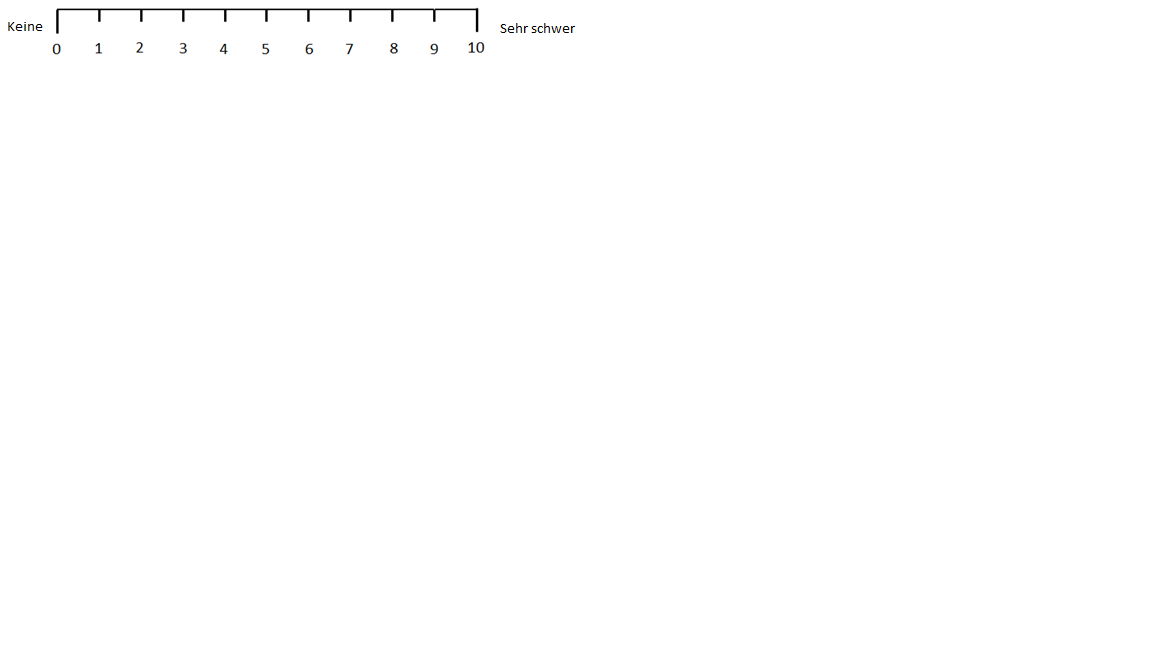
 Very important

**Information from the manufacturer**
Not at all important
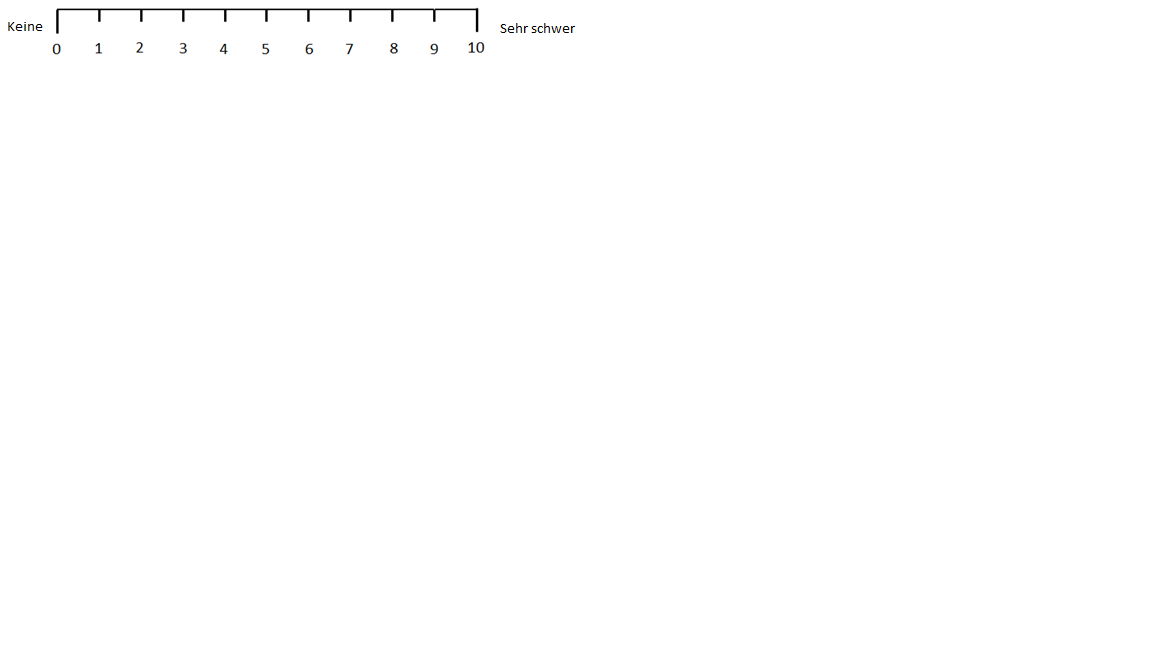
 Very important

**Personal experience**
Not at all important
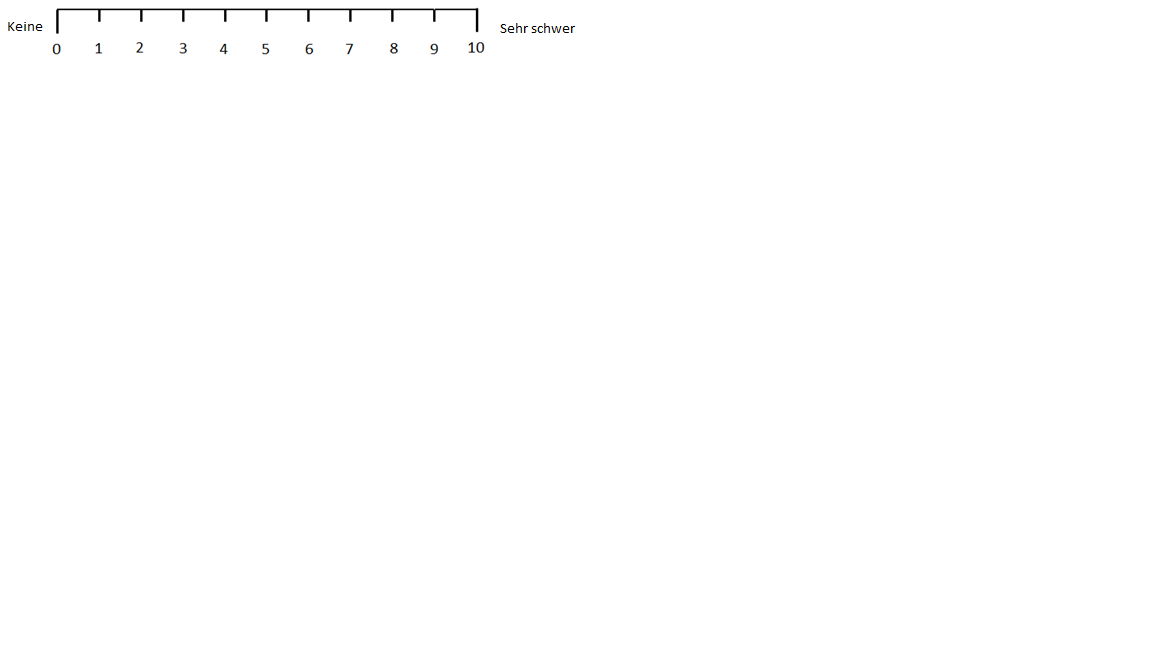
 Very important **Patient experience**
Not at all important
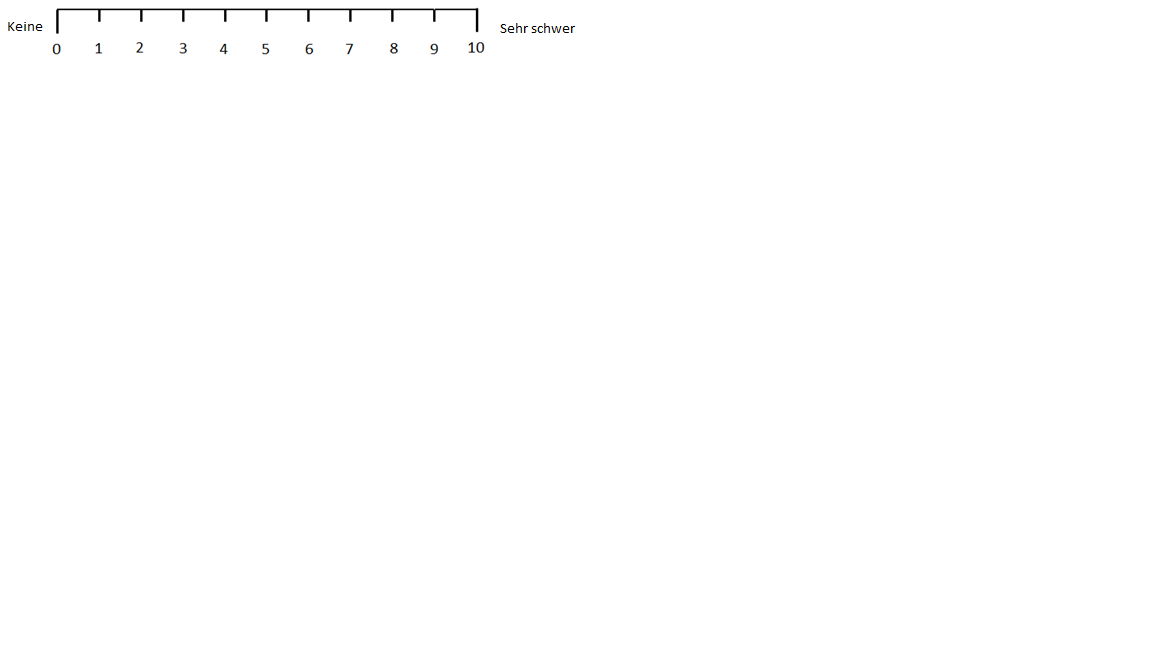
 Very important

1. **What impact could new adrenaline devices have at the community level**
   *Please rank the answers from the list below (0 No impact - 10 High impact)*

**Increased awareness of allergy and anaphylaxis in the community**
No impact
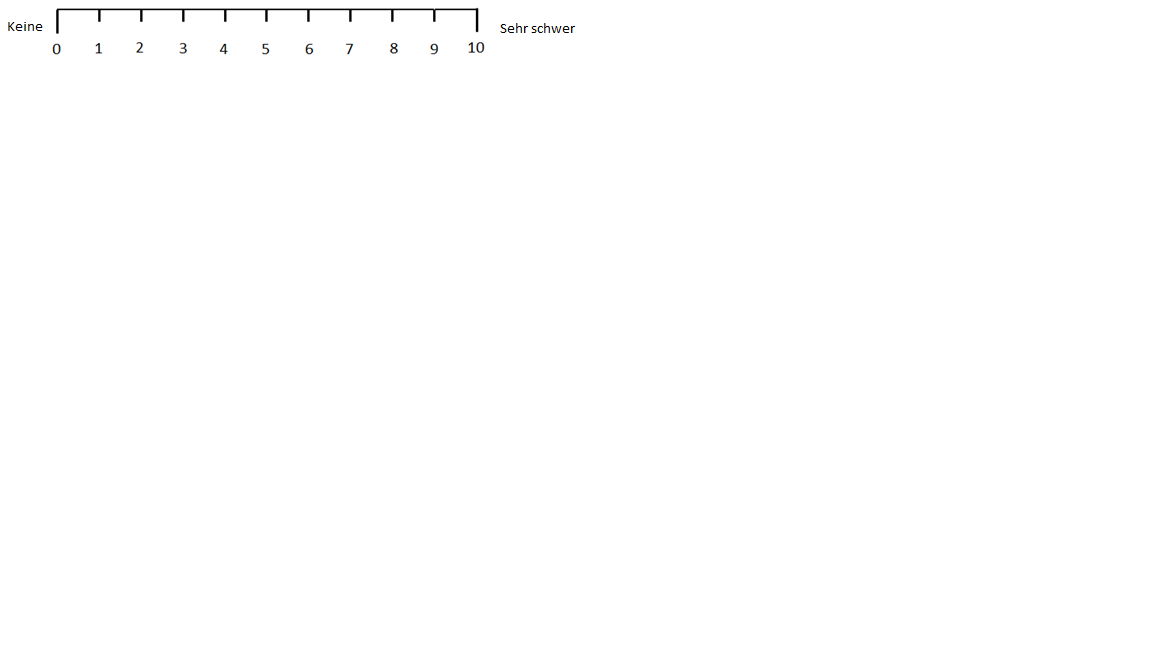
 High impact

**Increased use of adrenaline to treat anaphylaxis in the community at all**
No impact
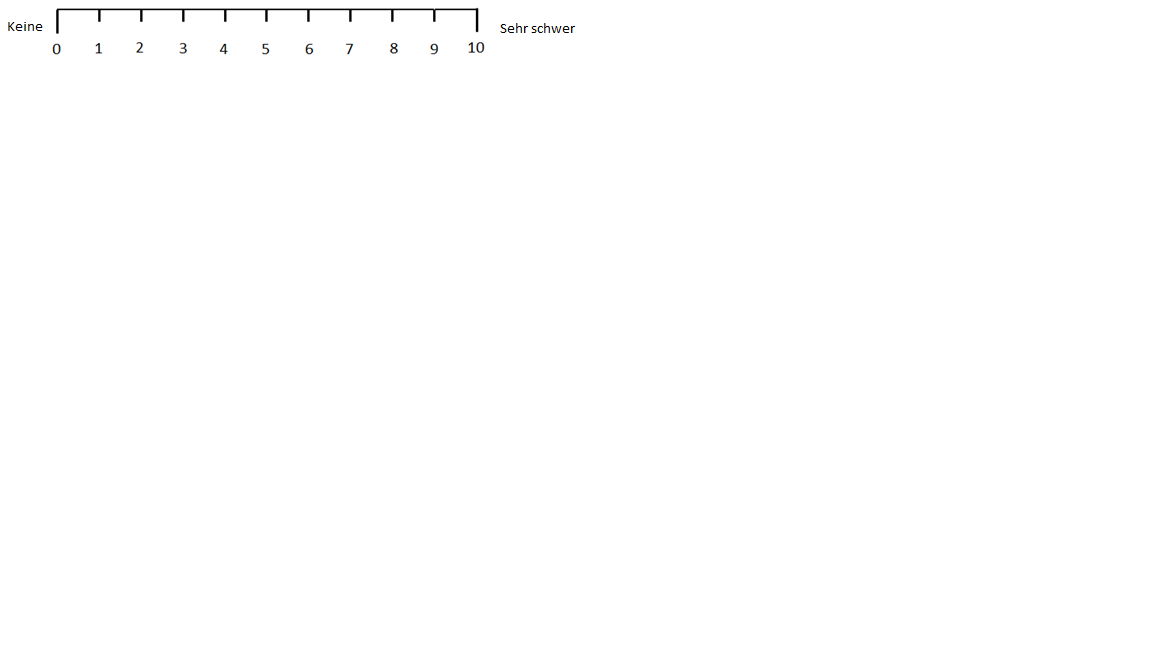
 High impact

**More prompt use of adrenaline in patients with severe anaphylaxis**
No impact
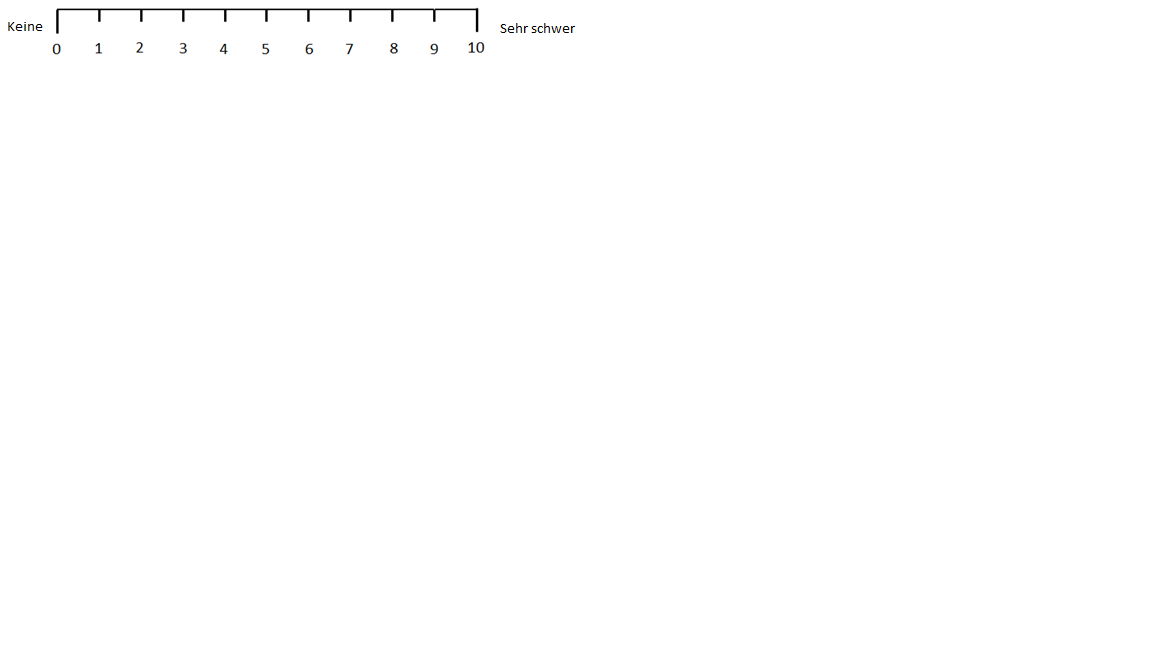
 High impact

**More prompt use of adrenaline in patients with mild to moderate anaphylaxis**
No impact
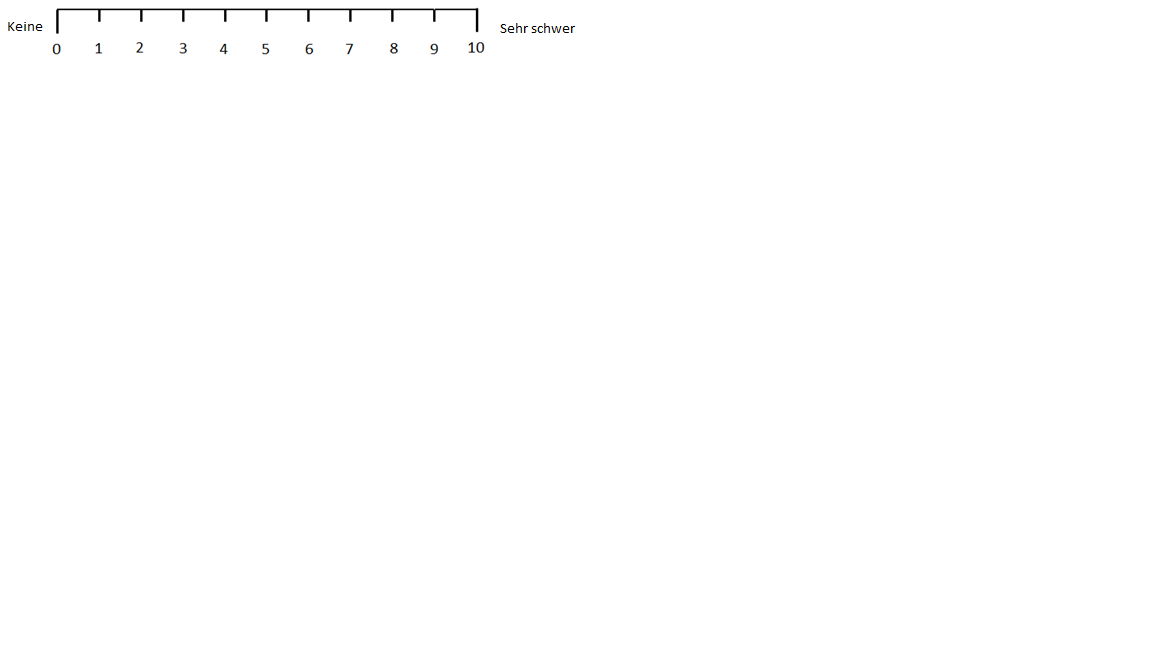
 High impact

**Wider access to adrenaline in public spaces**
No impact
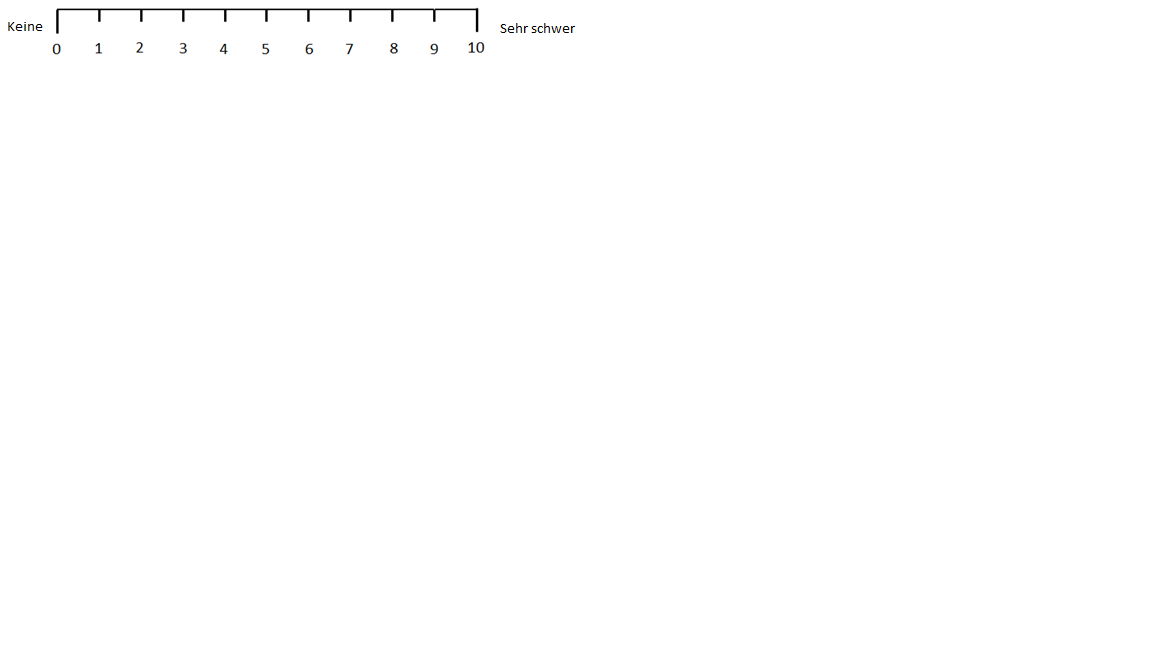
 High impact

**Wider access to adrenaline in schools**
No impact
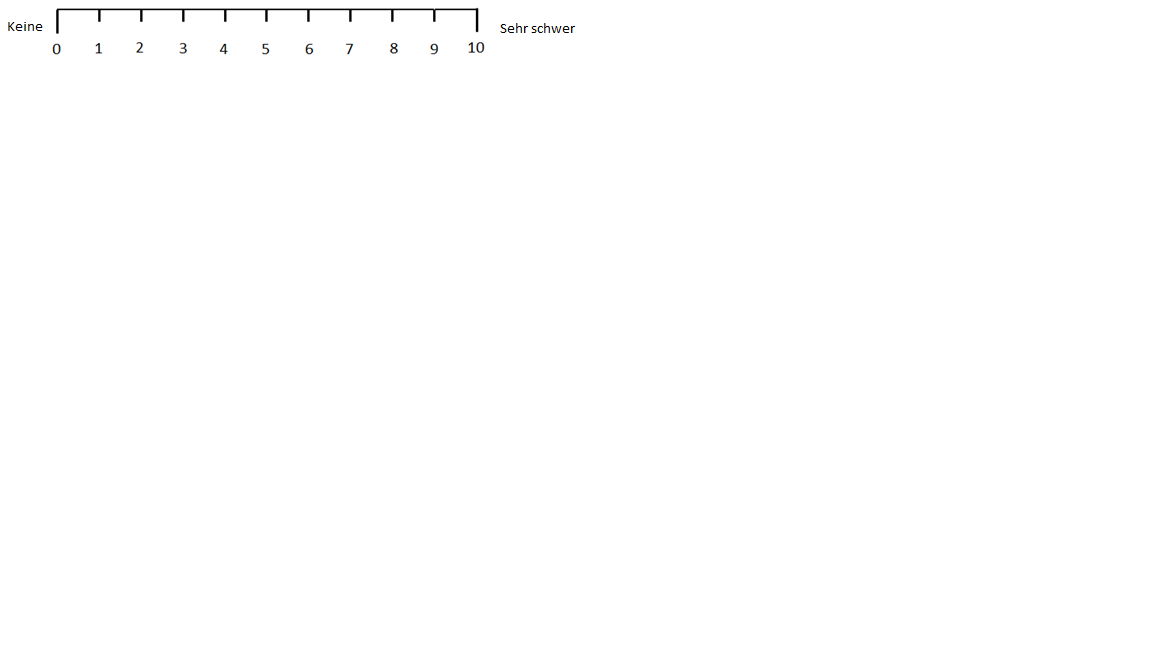
 High impact

**Improved global availability of adrenaline**
No impact
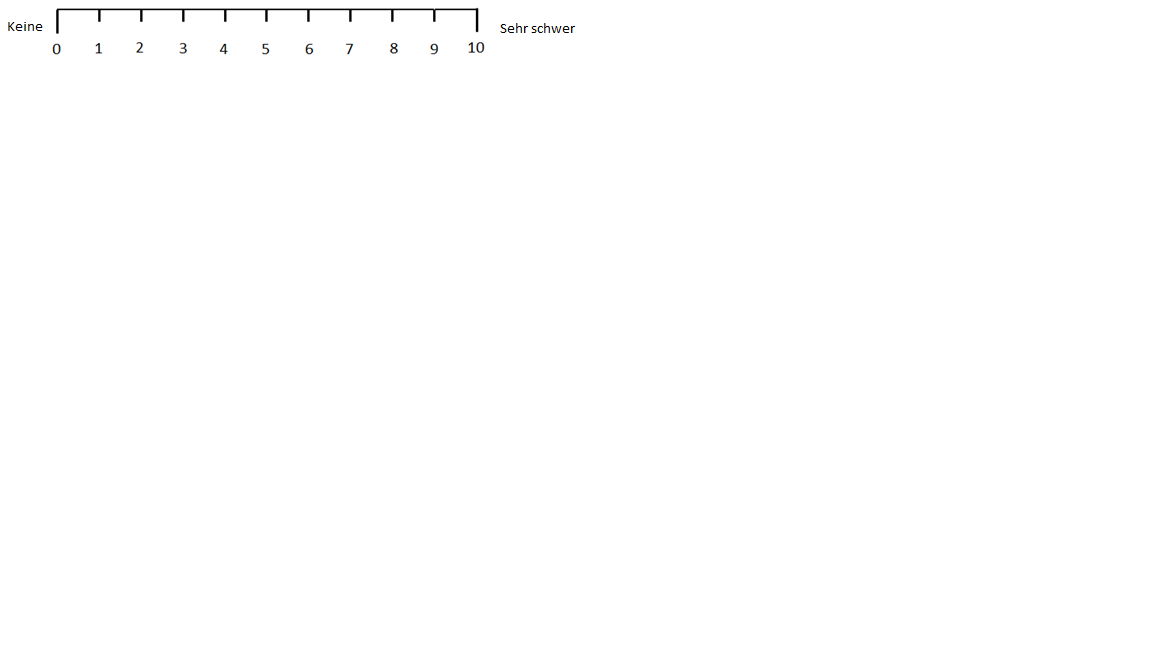
 High impact

**Other response (please specify): ___________**
No impact
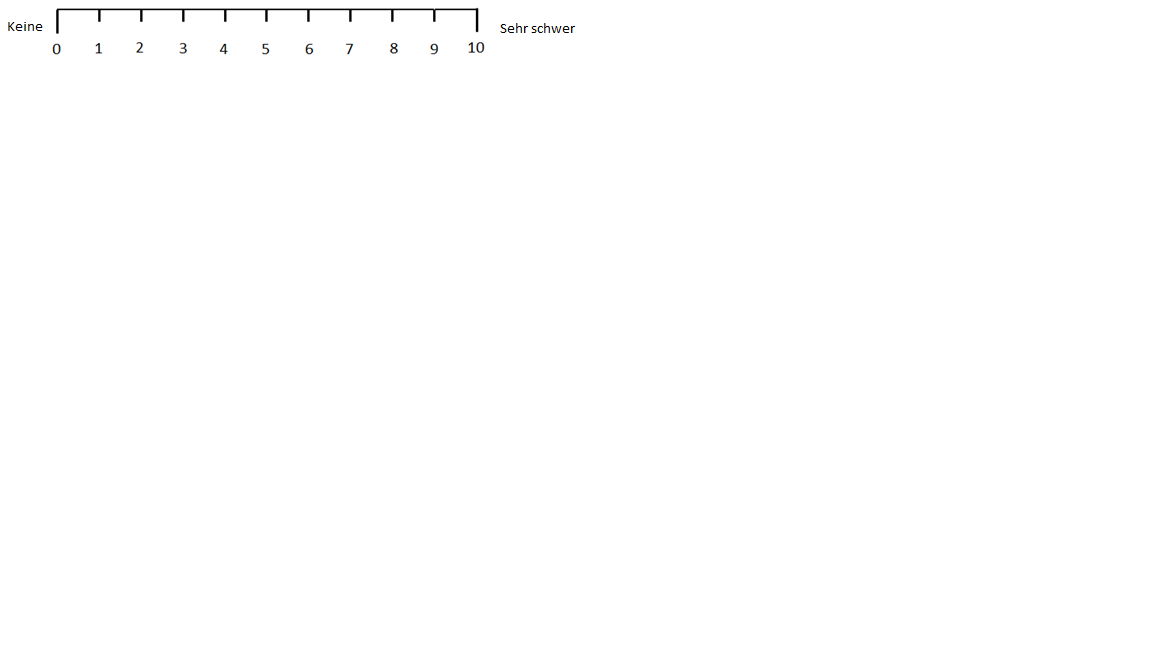
 High impact

**Thank you for your participation!**

**Your Anaphylaxis Team**
